# Alleviation of Microglia Mediating Hippocampal Neuron Impairments and Depression‐Related Behaviors by Urolithin B via the SIRT1‐FOXO1 Pathway

**DOI:** 10.1111/cns.70379

**Published:** 2025-04-16

**Authors:** Cuilan Liu, Di Zhao, Guoxing Yu, HengWei Du, Lihong Xu, Yifan Cao, Minghu Cui, Wentao Wang, Dan Wang, Jing Liu, Fantao Meng, Fengai Hu, Wei Li, Jing Du, Chen Li

**Affiliations:** ^1^ Department of Rehabilitation Medicine Binzhou Medical University Hospital Binzhou Shandong China; ^2^ Medical Research Center Binzhou Medical University Hospital Binzhou Shandong China; ^3^ Department of Psychology Binzhou Medical University Hospital Binzhou Shandong China

**Keywords:** cytotoxicity, depression, FOXO1, neuroinflammation, SIRT1, urolithin B

## Abstract

**Aims:**

Conventional antidepressants exhibit limited efficacy and delayed onset. This study aimed to elucidate the antidepressant effects of urolithin B (UB) and its regulatory role in microglia‐mediated hippocampal neuronal dysfunction.

**Methods:**

The mouse model of depression was established using both chronic unpredicted stress (CUS) and lipopolysaccharide (LPS) injection. The therapeutic efficacy of UB was assessed through behavioral paradigms. The microglia activation, cellular cytotoxicity and apoptosis levels, and underlying molecular mechanisms were delineated utilizing proteomics analysis, immunofluorescence staining, real‐time PCR and Western blotting.

**Results:**

UB efficiently alleviated depression‐related behaviors, accompanied by suppressed microglia activation, neuroinflammation, changes of classic activation (M1)/alternative activation (M2) polarization and recovered sirtuin‐1 (SIRT1) and forkhead box protein O1 (FOXO1) expression in the hippocampus. Additionally, UB reduced the cytotoxicity and apoptosis of HT22 cells and depression‐related phenotypes treated by the cellular supernatant from LPS‐incubated BV2 cells, which was mediated by the SIRT1‐FOXO1 pathway. The proteomics analysis of the cellular supernatant content revealed abundant secreting proteins among the LPS/UB application.

**Conclusion:**

This study confirmed that microglial SIRT1 mediates UB's antidepressant effects, positioning UB as a promising therapeutic candidate for depression by targeting neuroinflammatory pathways.

## Graphical abstract

UB exerts antidepressant activity and its modulation of microglia activation and neuroinflammation, balanced M1/M2 polarization via the SIRT1‐FOXO1 axis.

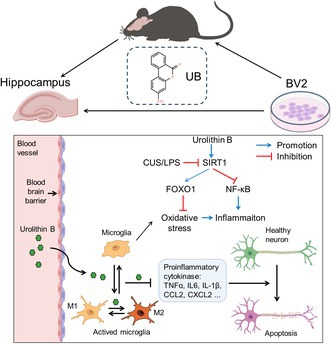

**Abbreviations**
A: Absorption
AAV: Adeno‐associated virus
Ac‐FOXO1: Acetylation‐FOXO1
Ac‐P65: Acetylation‐P65
ANOVA: Analysis of variance
ARG1: Arginase‐1
ATCC: American Type Culture Collection
BBB: American Type Culture Collection
BCA: American Type Culture Collection
BDNF: Brain‐derived neurotrophic factor
BP: Biological process
CC: Cell component
CD206: Mannose receptor C‐type 1
cDNA: Complementary DNA
CCK‐8: Cell counting kit
CNS: Central nervous system
CSF: Colony stimulating factor
CO_2_
: Carbon dioxide
CUS: Chronic unpredicted stress
CXC: C‐X‐C motif
DAPI
: 4',6‐Diamidino‐2‐phenylindole
DCFH‐DA: Dichloro‐dihydrofluorescein diacetate
DEP: Differentially expressed protein
DG: Dentate gyrus
DMEM: Dentate gyrus
DMSO: Dimethyl sulfoxide
EA: Ellagic acid
ERK: Extracellular signal‐regulated kinase
ET: Ellagitannin
FOXO1: Forkhead box protein O1
FST: Forced swimming test
FUST: Female urine sniffing test
GO: Gene ontology
HPLC: High performance liquid chromatography
KEGG: Kyoto encyclopedia of genes and genomes
Iba1: Ionized calcium‐binding adapter molecule 1
IDO: Indoleamine 2,3‐dioxygenase
IL‐6: Interleukin‐6
LPS: Lipopolysaccharide
M1: Classic activation
M2: Alternative activation
MAO‐A: Monoamine oxidase A
MAPK: Mitogen‐activated protein kinases
MDD: Major depressive disorder
MF: Molecular function
mPFC: Medial prefrontal cortex
NAD^+^
: Nicotinamide adenine dinucleotide
NC: Non‐specific control
RIPA: Radio immunoprecipitation assay
RNA: Ribonucleic acid
ROS: Reactive oxygen species
RP: Reversed phase
RT–PCR: Real‐time–PCR
PBS: Phosphate‐buffered saline
PCR: Polymerase chain reaction
PD: Parkinson's disease
PI: Propidium iodide
SCUS: subchronic unpredictable stress
SDS‐PAGE: Sodium dodecyl sulfate‐polyacrylamide gel electrophoresis
SIRT1: Sirtuin‐1
SPT: Sucrose preference test
SYBR: Synergetic binding reagent
TMT: Tandem mass tags
TNF‐ɑ: Tumor necrosis factor‐α
UA: Urolithin A
UB: Urolithin B
UC: Urolithin C
UD: Urolithin D

## 1 Introduction

As a common public health problem with a rising incidence rate, major depressive disorder (MDD) has seriously burdened both patients and society [1]. The estimated average lifetime prevalence of major depression is 11%–15% [2], and its recurrence ranges as high as 75%–90% [3]. Conventional antidepressants are ineffective in at least one‐third of patients and take several weeks or longer to achieve an effect [4]. Thus, novel strategies and efforts are needed to overcome treatment‐resistant depression.

Urolithins are the gut microbiota metabolites of ellagic acid (EA) and ellagitannins (ETs), including of urolithin A (UA), urolithin B (UB), urolithin C (UC) and urolithin D (UD) [5]. A wide variety of plant foods (e.g., berries and pomegranates) is an excellent source of EA and ET. Although the bioavailability of EA and ET in vivo is too low to be detected in systemic circulation and peripheral tissues, the metabolite urolithins can be absorbed and utilized, and the concentration can reach the micromolar level [6]. In addition, unlike ET and EA, urolithins are able to pass through the blood–brain barrier (BBB) to reach brain lesions [7, 8]. UB has been proven to be related to generating a neuroprotective effect in Parkinson's disease [9], cerebral ischemia [10], and cognitive deficits [11] and acts on various biological actions, including anti‐inflammatory [12, 13], anti‐tumor [14], and antioxidant [13, 15]. Studies have reported that UB reduces the inflammatory response of BV2 microglia stimulated by LPS, mainly by decreasing the inhibitor of kappa‐B ɑ (IκBɑ) phosphorylative level and inhibiting nuclear factor kappa‐B (NF‐κB) activity, protein kinase B (AKT), and mitogen‐activated protein kinases (MAPKs) signaling pathways and enhancing the phosphorylative level of AMP‐activated protein kinase (AMPK) [16, 17].

Neuroinflammatory processes have been implicated as a critical pathophysiology of MDD [18]. Microglia are the major resident immunological cells in the central nervous system (CNS) and play a key contribution in depression. The functions of microglia include regulation of CNS homeostasis and modulation of inflammatory response, synaptic plasticity, and neural network development [19]. Microglia carry out macrophage‐like morphology and function, and can polarize to the M1 classic activation or M2 alternative activation phenotype under appropriate circumstances [20]. When suffering from infection, damage, or stress, microglia turn to the M1 polarization type and secrete inflammatory factors to the extracellular environment, including interleukin‐6 (IL‐6), reactive oxygen species (ROS), interleukin‐1β (IL‐1β), and tumor necrosis factor‐α (TNF‐α), to engage in proinflammatory activity, which may damage surrounding neuronal cells [21, 22]. Anti‐inflammatory M2 microglia generally produce anti‐inflammatory mediators, such as arginase‐1 (ARG1), transforming growth factor‐β1 (TGF‐β1), and mannose receptor C‐type 1 (CD206) to promote phagocytosis of misfolded synthetic proteins and cell particles, tissue restoration, and neuronal survival [19, 21]. In vitro experiments demonstrated that conditioned media from LPS‐challenged microglia induced apoptosis of hippocampal neuroblasts, mediated by the secretion of IL‐6 and TNF‐α into the medium [23, 24]. The hippocampal neurogenesis and neuron growth are inhibited by microglia‐derived IL‐1 [25, 26]. The microglia activation induces the suppression of neurogenesis and neuroplasticity, which are considered important mechanisms of depression [19]. Besides, activation of indoleamine 2,3‐dioxygenase (IDO) in microglia is important for the development of depression [27]. Therefore, targeting microglia‐related inflammation may provide therapeutic benefits in treating depression.

SIRT1 is a type of the nicotinamide adenine dinucleotide (NAD^+^)‐dependent sirtuin deacetylases. It can deacetylate numerous substrates, such as transcription factors, histones, and many other proteins [28], thereby impacting various biological processes, including inflammation [29], oxidative stress [30], metabolism [6], and senescence [15]. Studies of MDD patients and animal models have revealed the essential role of SIRT1 in depression [31, 32]. Additionally, it acts as a vital function in the pathogenesis of depression by impacting inflammation, neurogenesis, circadian rhythm, and brain‐derived neurotrophic factor (BDNF), extracellular signal‐regulated kinase (ERK), and other signal pathways [17]. There is a crosstalk between SIRT1 and NF‐κB to regulate ROS and the inflammation process. NF‐κB signaling and inflammation can negatively impact the activity of SIRT1; conversely, SIRT1 inhibits NF‐κB activation [30, 33]. FOXO1, which belongs to the FOXO family, is one of the downstream genes of SIRT1. FOXO1 performs essential functions by modulating downstream genes, for example, immune regulators and anti‐oxidative stress enzymes [34]. FOXO1 is highly expressed in brain regions associated with mood and stress regulation, and mice deficient in FOXO1 exhibit depression‐like behavior [35], suggesting that FOXO1 may take part in the pathological process of depression. The acetylation or transcriptional levels of FOXO1 can be modulated via SIRT1‐mediated deacetylation [36]. Existing investigations have demonstrated that the SIRT1‐FOXO1 pathway exerts a wide range of functions and has antidepressant effects by encouraging vascular and neural regeneration in the hippocampus [29]. Specifically, SIRT1‐FOXO1 was discovered to ameliorate depression‐related phenotypes in Parkinson's disease (PD) mouse models by regulating the transcription of monoamine oxidase A (MAO‐A) [37].

In the present research, we investigated the antidepressant functions of UB on CUS and LPS‐induced depression behaviors. Then we investigated the potential mechanisms of anti‐neuroinflammatory and anti‐oxidative actions of UB on LPS‐treated BV2 microglia cells and evaluated its therapeutic activities against the cytotoxicity of cellular supernatant from LPS‐incubated BV2 cells in vivo and in vitro. Finally, we explored the impact of SIRT1 knockout on the antidepressant effect of UB.

## 2 Materials and Methods

### 2.1 Animals

Seven‐week‐old male C57BL/6 J mice were purchased from Jinan Pengyue Experimental Animal Breeding Company (Jinan, China). Three to five animal groups were raised together under a 12:12 h light/dark cycle at a temperature of 23°C ± 2°C during which they had unfettered access to both water and food. The research protocol was approved by the Institutional Animal Care and Use Committee of the Binzhou Medical University Hospital and in accordance with the National Institutes of Health Guide for the Care and Use of Laboratory Animals (NIH Publications No. 8023, revised 1978).

### 2.2 Chronic Unpredictable Stress (CUS)

The CUS procedure was carried out following the previously outlined methodology [35, 38, 39]. Briefly, the paradigm involved a variety of stress stimuli (including restraint stress for 1 h, tail pinch for 20 min, shaking and crowding for 1 h, cold swim for 5 min at 8°C, wet bedding for 4 h, constant light for 24 h, and electric shock for 10 min) applied randomly across daily activities over 14 days. To assess stress sensitivity, a brief subchronic unpredictable stress (SCUS) consisting of the initial 7 days of diverse stressors was utilized.

### 2.3 LPS Induced Depression Model and UB Treatment

For the depression mouse model with LPS exposure, LPS (L2630, Sigma, USA) dissolved in 0.9% saline vehicle was administered intraperitoneally (i.p.) at a specified dosage of 1 mg/kg [40]. An identical volume of vehicle was injected as control administration. For UB treatment, mice received intraperitoneal injection of 10 mg/kg UB (HY‐126307, MedChemExpress, USA) for 7 days before LPS injection.

### 2.4 Knockout of SIRT1 and Stereotaxic Surgery

The adeno‐associated virus (AAV), containing a titer higher than 1 × 10^12^ vg/mL, was packaged with the oligonucleotide AGTGAGACCAGTAGCACTAAT targeted SIRT1 with an independent U6 promoter (Hanbio, Shanghai, China), and the none‐specific control (NC) was the oligonucleotide TTCTCCGAACGTGTCACGT. BrainVTACo. Ltd. (Wuhan, China) provided the following AAVs: AAV2/9‐CX3CR1‐Cre (AAV‐CX3CR1‐Cre) and AAV2/9‐hSyn‐DIO‐GFP (AAV‐DIO‐GFP). As previously described, intra‐dentate gyrus (DG) infection via virus injection was performed (coordinates: anteroposoterior [AP] = −2.1 mm, mediolateral [ML] = ±2.1 mm, dorsoventral [DV] = −2.3 mm from the bregma) [39]. Briefly, a bilateral injection of a volume of 0.50 μL AAV vector into each side of the DG was performed at a speed of 0.10 μL/min using a 33‐gauge stainless steel injector connected to a UMP3 micro syringe pump (World Precision Instruments, USA). After injection, we waited 5 min for spread and stoppage of reflux. Behavioral testing was done on day 21 post‐injection.

### 2.5 Sucrose Preference Test

Before testing, two same bottles full of drinking water were placed in the cage, and the mice were free to drink from two sides to habituate for at least 7 days. The mice were kept singly in cages and given free access to either water or a 1% sugar solution for 2 h of darkness. The consumption of water and sugar water was weighed separately, and the preference for the sucrose solution: (sucrose preference) = [(sucrose consumption)/(sucrose consumption + water consumption)] × 100% was calculated [41].

### 2.6 Forced Swimming Test

For the forced swimming experiment, we performed exactly as formerly reported [39, 42]. A mouse was separately located in a transparent Plexiglas cylinder (internal diameter 10 cm, height 25 cm) stuffed with 15 cm–deep water at a temperature of 24°C for 6 min. The entire process was documented via high‐definition video. An observer blinded to the animal group measured the period of immobility in the final 4 min.

### 2.7 Locomotor Activity

SuperFlex Fusion open field cages (40 cm × 40 cm × 30 cm, Omnitech Electronics Inc., USA) were used to assay locomotor behavior. Mice were placed in the cages where they explored a novel environment for 30 min in a well‐lit environment. Total movement distance and travel were determined using data obtained from photo sensors hooked up to the cages via Fusion software (Omnitech Electronics Inc.) [43].

### 2.8 Cell Cultures and Drug Treatment

Mouse microglia cells BV2 and mouse HT22 hippocampal neuron cells were purchased from the American Type Culture Collection (ATCC) (ATCC, USA) and nurtured in Dulbecco's Modified Eagle's Medium (DMEM) containing 10% fetal bovine serum and 100 U/mL of penicillin/streptomycin (Gibco, USA) at 37°C under a 5% carbon dioxide (CO_2_) humidified incubator. Various doses of UB (10 μmol/L, 20 μmol/L, and 40 μmol/L) were treated to the BV2 cells for a period of 2 h before co‐treatment with 0.5 μg/mL LPS for 24 h. The control group was administered an equal amount of vehicle. To examine whether SIRT1 and FOXO1 mediated the anti‐inflammatory activity of UB, inhibitors targeting SIRT1 or FOXO1, EX‐527 (40 μmol/L, dissolved in DMSO, HY‐15452, MedChemExpress) or AS1842856 (1 μmol/L, dissolved in DMSO, HY‐100596, MedChemExpress), were administered 1 h prior to UB exposure and subsequently with LPS stimulation. After drug treatment, the supernatant medium of BV2 cells was collected as the conditional medium to treat HT22 cells for 24 h.

### 2.9 Real‐Time PCR Quantitation of Gene Expression

For the mouse brain tissue, Trizol (15596026, Invitrogen, USA) was utilized to isolate the total RNA. Extraction and purification of the total RNA of the cell lines were performed using the Total RNA Kit (#R6834; OMEGA Bio‐teck, USA) according to the instructions from the manufacturer. Following this, reverse transcription was accomplished using 5 × HiScript II QRT SuperMix (Vazyme, China). The cDNA was detected using AceQ qPCR SYBR Green Master Mix (Vazyme) according to the operating instructions. The primers are displayed in Table S1. The 2^−ΔΔCT^ formula was employed for calculating the qualified expression levels [44].

### 2.10 Western Blot Assay

Following cervical dislocation, the hippocampus and medial prefrontal cortex (mPFC) tissues of mice were isolated and harvested rapidly on ice. For the BV2 and HT22 cells, cells were collected using centrifugation. Homogenized tissue or cell extract was obtained by using radio immunoprecipitation assay (RIPA) lysis buffer comprising protease inhibitors and a 1 × PhosSTOP phosphatase inhibitor cocktail (Roche Applied Science, Germany). The concentration of protein was determined using a BCA protein assay kit (Vazyme). The proteins were separated by an SDS‐PAGE system and transferred to a polyvinylidene fluoride (PVDF) membrane (Millipore, USA) in a Trans‐Blot SD semidry transfer cell (Bio‐Rad, USA). The transferred membranes were incubated for one night with the primary antibodies at 4°C. Following probing with secondary antibodies, the PVDF membranes were detected by the Odyssey infrared imaging system (Li‐COR Biosciences, USA). The primary antibodies employed in this study were as follows: rabbit anti‐SIRT1 (Sigma‐Aldrich, 07‐131), rabbit anti‐FOXO1 (CST, 2880S), mouse anti‐β‐actin (CST, 3700S), rabbit anti‐P65 (CST, 8242T), rabbit anti‐phospho‐P65 (CST, 3031S), ARG1 (BOSTER, A01106), CD206 (BOSTER, A02285‐2), CD86 (BOSTER, A00220‐4), iNOS (BOSTER, BA0362), ERK (CST, 4695), p‐ERK (CST, 4370), rabbit anti‐P38 (CST, 9212S), phospho‐P38 (CST, 9215), JNK (CST, 9251), phospho‐JNK (CST, 9252), rabbit anti‐AMPK (CST, 5831S), rabbit anti‐phospho‐AMPK (CST, 2535S), rabbit anti‐Bax (CST, 5023T), mouse anti‐Bcl‐2 (CST, 15071T), mouse anti‐IDO (Proteintech, 66528‐1‐Ig), rabbit anti‐Ac‐FOXO1 (Invitrogen, PA5‐104560), rabbit anti‐Ac‐P65 (Abcam, AB19870).

### 2.11 Immunofluorescence Histochemistry

Mice were perfused, and the whole brains from mice were fixed post‐embedding overnight in 4% paraformaldehyde, then dehydrated with a 30% sucrose solution and sliced coronally at 40 μm thickness. For cells, BV2 cells were fixed with 4% paraformaldehyde. Afterwards, cells and the sections were washed in phosphate‐buffered saline (PBS) and permeabilized with immunoblocking buffer for 1 h at room temperature, incubated with Iba1 antibody (1:400, ab5076, Abcam, UK), SIRT1 antibody (1: 400, Abclonal, A0230), and FOXO1 antibody (1:1000, CST, 2880). After washing with PBS, the sections were probed with Alexa Fluor 546 goat anti‐rabbit IgG antibody (1:400, A11035, Invitrogen, USA), Alexa Fluor 594 goat anti‐rabbit IgG antibody (1:400, A11012, Invitrogen), Alexa Fluor 546 donkey anti‐goat IgG antibody (1:400, A11045, Invitrogen), and Alexa Fluor 488 donkey anti‐rabbit IgG antibody (1:400, A21206, Invitrogen) for 1 h at room temperature. Following washes in PBS, the sections were incubated with DAPI (3 μg/mL) (Beyotime Biotechnology, China) for 5 min to stain the nuclei. Last, the well‐stained sections were washed with PBS and located on poly‐lysine‐coated glass slides with coverslips and captured using confocal microscopy (Olympus, Japan) under identical conditions [45]. We analyzed immunofluorescence intensity in the cells using ImageJ (http://imagej.nih.gov/ij/) as per previous description [46].

### 2.12 CCK8

The cells were counted and seeded with 3000 cells per well in 96‐well plates. Multiple concentrations of drugs were administered to the cells, and the CCK‐8 assay (MedChemExpress) was performed at 24 h following the manufacturer's protocol. The absorption (A) was assessed with a SpectraMax M5 microplate reader (Molecular Devices, USA) at 450 nm. The relative A was normalized to the control. Experiments were performed in triplicates. Cell viability = (A value of experimental group‐A value of blank group)/(A value of control group‐A value of blank group) × 100%.

### 2.13 ROS

Detection of intracellular ROS levels was achieved through dichloro‐dihydro‐fluorescein diacetate (DCFH‐DA) fluorescence assay kit (S0033S, Beyotime Biotechnology) according to the guidelines provided by the manufacturer. In short, cells underwent incubation in newly prepared DMEM medium added with 10 μM DCFH‐DA at 37°C for 30 min. Subsequently, the cells were rinsed twice using PBS and observed; images were captured under a fluorescence microscope.

### 2.14 Live and Dead Cell Assays

The HT22 cells were first populated into 6‐well plates to be incubated with conditional medium (CM) for 24 h. After adding a staining mixture comprising 2 μmol/L Calcein AM and 4.5 μmol/L PI, the cells were incubated at room temperature for 15 min. Following washes with PBS, the cells were photographed by fluorescence microscope, and the dead cell ratio was computed as follows: dead cell rate = dead cell number/(live cell number + dead cell number) × 100%.

### 2.15 Quantitative Proteomics Analysis

Dishes containing adherent BV2 cells were changed to culture media free of serum. UB and LPS were added successively and continued to be cultured for 24 h. Cell culture was collected using centrifugation and quickly frozen in liquid nitrogen. The supernatant contains all proteins present in the sample, and the BCA method is utilized to ascertain the protein concentration. Analysis of the proteomic data was conducted using Shanghai Luming biological technology co. LTD (Shanghai, China). The samples were digested with trypsin and labeled with TMT/iTRAQ. An 1100 high‐performance liquid chromatography (HPLC) System (Agilent) was used to perform reversed‐phase (RP) separation with an Agilent Zorbax Extend RP column (5 μm, 150 mm × 2.1 mm). The RP gradient utilized a mobile phase composed of mobile phase A containing 2% acetonitrile in HPLC water and mobile phase B containing 98% acetonitrile in HPLC water. Lyophilization was performed on the isolated peptides to prepare them for mass spectrometry. The complete set of analyses was conducted using the Q Exactive HF mass spectrometer fitted with a Nanospray Flex source (all from ThermoFisher, USA). The entire set of raw data was searched with proteomeDiscoverer 2.4.1.15 (ThermoFisher) against the Uniprot *Mus Musculus* database. Database searches were conducted with trypsin digestion specificity, and alkylation of cysteine was included as a fixed modification in the search parameters. For protein quantification, the TMT/iTRAQ method was selected. A global false discovery rate (FDR) threshold of 0.01 was applied, and protein groups were considered for quantification only if they contained at least one unique peptide. We defined the differentially expressed proteins (DEPs) by setting the thresholds of fold change (> 1.5 or < 1/1.5) and a *p*‐value < 0.05. Gene ontology (GO) (http://www.blast2go.com/b2ghome; http://geneontology.org/) and the kyoto encyclopedia of genes and genomes (KEGG) pathway (http://www.genome.jp/kegg/) annotations were assigned to all identified proteins. Furthermore, DEPs were used for the GO and KEGG enrichment analysis. String (https://string‐db.org/) database was employed to analyze the protein–protein interactome.

### 2.16 Statistical Analyses

Data analysis was carried out with GraphPad Prism 9. Use of the Shapiro–Wilk and *F* test(s) for checking the assumption of normality and equality of variances was conducted. Multiple comparisons of normally distributed data were conducted with one‐way analysis of variance (ANOVA) followed by the Sidak post hoc test or two‐way ANOVA followed by Tukey's test. The *p* < 0.05 attained statistical significance. The experiments in vitro were conducted in three independent replicates. The data are all expressed in the form of mean ± standard error.

## 3 Results

### 3.1 Improvement in Depression‐Like Behaviors, Microglia Activation, Neuroinflammation, Balance of Microglial M1/M2 Polarization, and Abnormal SIRT1 and FOXO1 Expression due to UB in the CUS‐Induced Model

**FIGURE 1:**
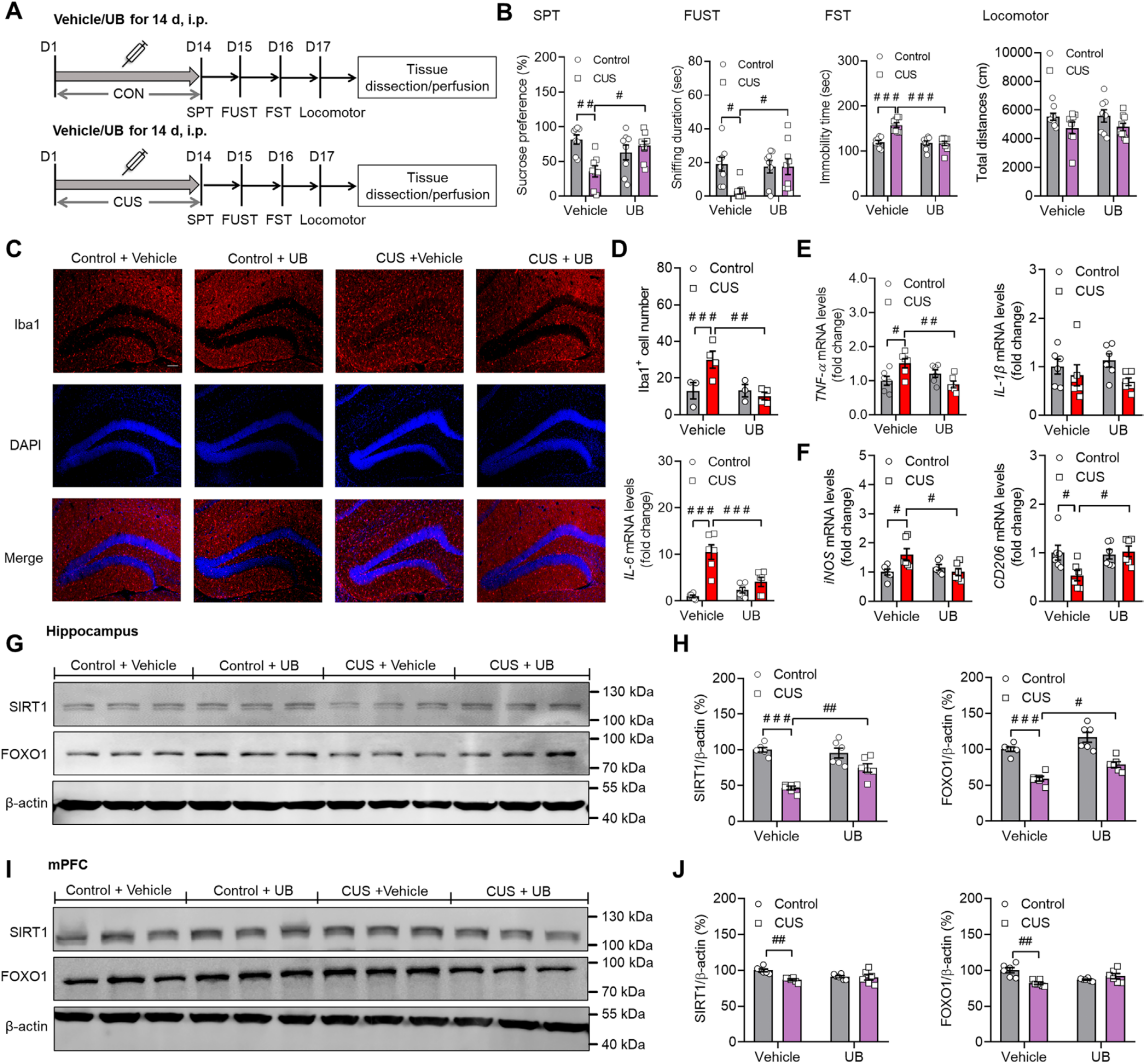
UB ameliorates CUS‐induced depressive behaviors, neuroinflammation and abnormal expression of SIRT1 and FOXO1 in hippocampus. (A) Schematic representation of the CUS procedure and treatments in mice. SPT, sucrose preference test; FST, forced swimming test; FUST, Female urine sniffing test; i.p., intraperitoneal injection. (B) SPT, FST, FUST and locomotor test. (C) Immunofluorescence micrographs of microglia immunostaining for Iba1. Iba1^+^ microglia are colored red, and nuclei are stained with DAPI (blue). Scale bar, 100 μm. (D) Quantification of Iba1 positive cells. (E) Quantification of the relative mRNA levels of TNF‐ɑ, IL‐1β and IL‐6 in hippocampus. (F) Quantification of the relative mRNA levels of iNOS and CD206 in hippocampus. Representative western blot bands and relative protein levels of SIRT1 and FOXO1 in (G and H) hippocampus and in (I and J) mPFC. *n* = 6–9 per group. ^*#*^ *p* < 0.05, ^*##*^ *p* < 0.01, ^*###*^ *p* < 0.001 versus the Control + Vehicle group or CUS + Vehicle group.

In order to evaluate the protective and therapeutic activity of UB in depression, we first screened the effective dose of UB. The wild‐type mice were subjected to the CUS process with different concentrations of UB (5 mg/kg and10 mg/kg) treatment once per day (Figure S1A), and the results in SPT reveal that only the higher dose of UB (10 mg/kg) reversed the CUS‐induced reduction in sucrose intake (*p* = 0.0021 and *p* = 0.0374; *F* (3, 29) = 6.5780, *p* = 0.0031). In the forced swim test, compared to the control group, the immobility time of mice in the CUS group was significantly increased (*p* = 0.0092), while UB treatment reversed this phenotype at 10 mg/kg (*p* = 0.0030) (*F* (3, 29) = 6.4720, *p* = 0.0033) (Figure S1B). Therefore, we selected 10 mg/kg as the experimental dose in the following experiments. Next, multiple depression‐like behaviors were tested after UB was injected into the mice once per day during the CUS process (Figure 1A). In SPT, the reduced sucrose preference in CUS mice was reversed by UB treatment (*p* = 0.0024 and *p* = 0.0136) (Figure 1B; stress: *F* (1, 29) = 4.9520, *p* = 0.0340, treatment: *F* (1, 29) = 1.2830, *p* = 0.2666, interaction: *F* (1, 29) = 11.8400, *p* = 0.0018). The FST results revealed that CUS increased the immobility time of control mice (*p* < 0.0010), while the UB administrations significantly shortened it (*p* < 0.0010) (Figure 1B; stress: *F* (1, 29) = 14.1000, *p* < 0.0010, treatment: *F* (1, 29) = 18.6600, *p* < 0.0010, interaction: *F* (1, 29) = 15.460, *p* < 0.0010). In FUST, there was an obvious decrease in the sniffing time of CUS mice (*p* = 0.0313), which was restored by UB (*p* = 0.0480) (Figure 1B; stress: *F* (1, 29) = 4.3730, *p* = 0.0454, treatment: *F* (1, 29) = 2.9290, *p* = 0.097, interaction: *F* (1, 29) = 4.4610, *p* = 0.0434). The locomotor activity showed no difference among these groups (Figure 1B; stress: *F* (1, 29) = 5.0600, *p* = 0.0322, treatment: *F* (1, 29) = 0.0328, *p* = 0.8574, interaction: *F* (1, 29) = 0.0004, *p* = 0.9832).

As the microglia activation and neuroinflammation were the crucial contributors to depression [19]. We stained and calculated the Iba1 positive cells and observed that UB treatment attenuated the risen number of Iba1 positive cells that emerged from the CUS process (*p* = 0.0440 and 0.0118) (Figure 1C,D; stress: *F* (1, 10) = 3.3780, *p* = 0.0959, treatment: *F* (1, 10) = 6.7350, *p* = 0.0267, interaction: *F* (1, 10) = 6.7720, *p* = 0.0264). Furthermore, some key proinflammatory and anti‐inflammatory factors were measured, and the results demonstrated that there was a significant elevation of TNF‐α and IL‐6 for CUS treated mice (*p* = 0.0296 and *p* < 0.0010), which was recovered by UB (*p* = 0.0070 and *p* < 0.0010) (TNF‐α: stress: *F* (1, 20) = 0.6521, *p* = 0.4289, treatment: *F* (1, 20) = 3.0150, *p* = 0.0979, interaction: *F* (1, 29) = 12.2900, *p* = 0.0022; IL‐6: stress: *F* (1, 20) = 33.0700, *p* < 0.0010, treatment: *F* (1, 20) = 6.6010, *p* = 0.0183, interaction: *F* (1, 20) = 15.4000, *p* < 0.0010). The mRNA expression levels of IL‐1β, IL‐4, and IL‐10 showed no obvious changes among the groups (IL‐1β: stress: *F* (1, 20) = 4.0950, *p* = 0.0566, treatment: *F* (1, 20) = 0.0002, *p* = 0.9888, interaction: *F* (1, 20) = 0.7280, *p* = 0.4037; IL‐4: stress: *F* (1, 20) = 1.2620, *p* = 0.2746, treatment: *F* (1, 20) = 0.0002, *p* = 0.9987, interaction: *F* (1, 20) = 0.8295, *p* = 0.3733; IL‐10: stress: *F* (1, 20) = 0.3828, *p* = 0.5431, treatment: *F* (1, 20) = 0.0312, *p* = 0.8615, interaction: *F* (1, 20) = 0.1018, *p* = 0.7530) (Figures 1E and S1C).

The research highlights that the imbalance between microglial M1 and M2 phenotypes significantly influences the inflammatory state, which is associated with the pathology of psychiatric disorders [47, 48]. Next, we tested the typical markers of M1/M2 polarization and observed that there was an increase in iNOS mRNA levels (*p* = 0.0289), which was suppressed by UB application (*p* = 0.0290). The groups exhibited no comparable difference in the expression levels of CD86 (M1 marker) (iNOS: stress: *F* (1, 20) = 2.5090, *p* = 0.1289, treatment: *F* (1, 20) = 2.5030, *p* = 0.1293, interaction: *F* (1, 20) = 7.5420, *p* = 0.0124, CD86: stress: *F* (1, 20) = 12.4200, *p* = 0.0021, treatment: *F* (1, 20) = 1.1160, *p* = 0.3034, interaction: *F* (1, 20) = 0.1227, *p* = 0.7298). Nevertheless, CUS stimulation decreased the levels of CD206 and ARG1 (M2 marker) compared to the control mice (*p* = 0.0496 and *p* = 0.0414). UB application notably attenuated the decline of CD206 (*p* = 0.0366), with no changes in another marker ARG1 (*p* = 0.9652) (CD206: stress: *F* (1, 20) = 3.0190, *p* = 0.0977, treatment: *F* (1, 20) = 3.7810, *p* = 0.0660, interaction: *F* (1, 20) = 4.9590, *p* = 0.0376; ARG1: stress: *F* (1, 20) = 6.5570, *p* = 0.0186, treatment: *F* (1, 20) = 0.7488, *p* = 0.3971, interaction: *F* (1, 20) = 2.3340, *p* = 0.1423) (Figures 1F and S1D).

Next, we measured SIRT1 and FOXO1 expression levels in the hippocampus and mPFC of these mice and observed that CUS reduced the protein levels of SIRT1 (*p* < 0.0010) and FOXO1 (*p* < 0.0010) in the hippocampus (*p* < 0.0010), whereas UB reversed their decreased expressions (*p* = 0.0037 and *p* = 0.0286) (Figure 1G,H; SIRT1: stress: *F* (1, 20) = 55.1500, *p* < 0.0010, treatment: *F* (1, 20) = 5.5460, *p* = 0.0288, interaction: *F* (1, 20) = 10.7900, *p* = 0.0037; FOXO1: stress: *F* (1, 20) = 75.2600, *p* < 0.0010, treatment: *F* (1, 20) = 15.9800, *p* < 0.0010, interaction: *F* (1, 20) = 0.1144, *p* = 0.7388). Moreover, we observed that CUS reduced the protein levels of SIRT1 (*p* = 0.0043) and FOXO1 (*p* = 0.0011) in the mPFC. However, UB did not protect against their reduction in mPFC (*p* = 0.7234 and *p* = 0.0941) (Figure 1G,I; SIRT1: stress: *F* (1, 20) = 8.4380, *p* = 0.0088, treatment: *F* (1, 20) = 1.3320, *p* = 0.2621, interaction: *F* (1, 20) = 6.9550, *p* = 0.0158; FOXO1: stress: *F* (1, 20) = 5.3590, *p* = 0.0314, treatment: *F* (1, 20) = 0.3526, *p* = 0.5593, interaction: *F* (1, 20) = 16.8200, *p* < 0.0010).

### 3.2 UB Regulated Depression‐Like Behaviors, Neuroinflammation, Microglial M1/M2 Polarization, and SIRT1 and FOXO1 Expression in LPS‐Induced Mice

**FIGURE 2:**
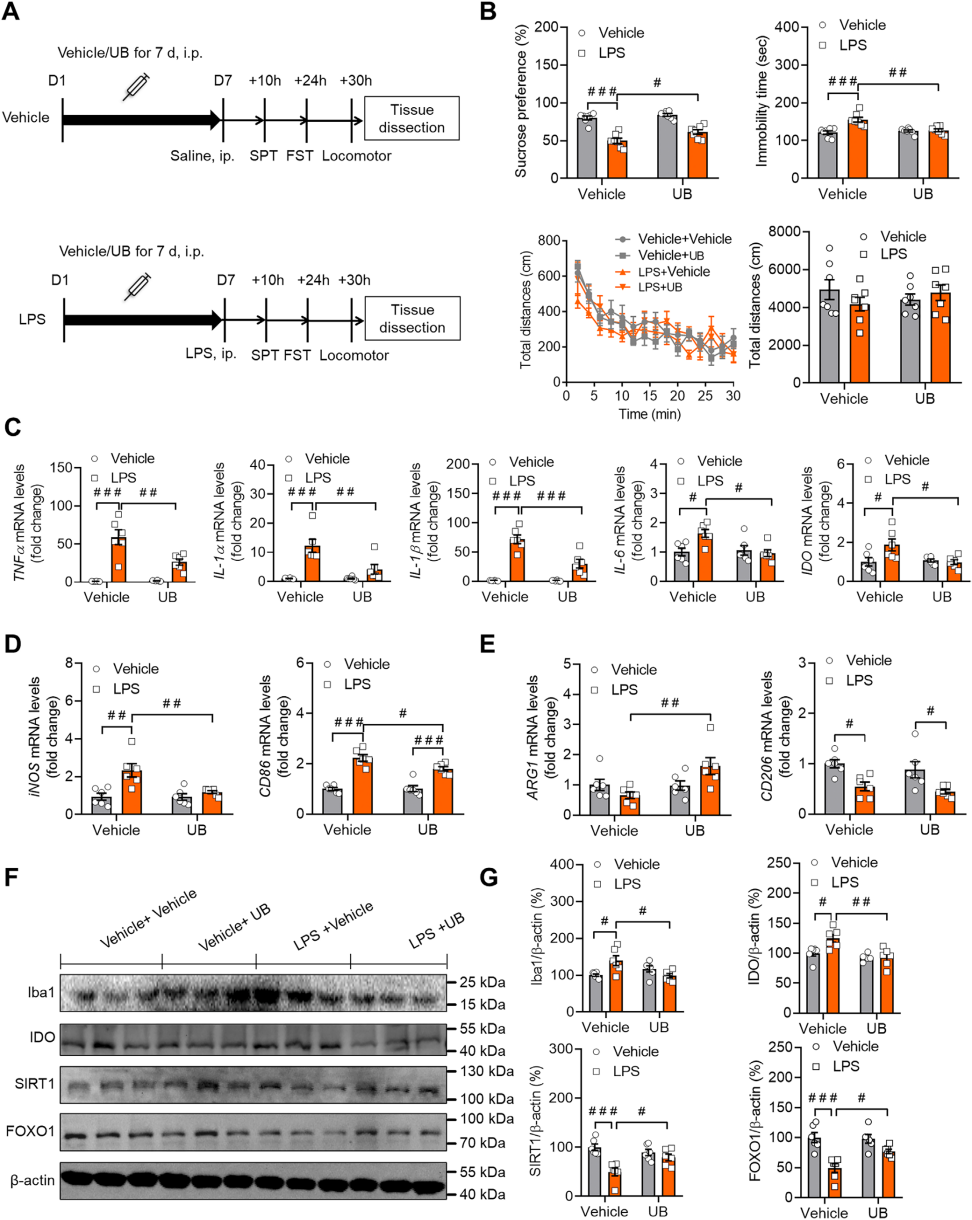
UB treatment ameliorates depression‐like behaviors and neuroinflammation and abnormal expression of SIRT1 and FOXO1 in the hippocampus of LPS‐exposed mice. (A) Scheme of the experimental procedure. (B) SPT, FST, and locomotor tests. (C) Relative mRNA levels of TNF‐ɑ, IL‐1ɑ, IL‐1β, IL‐6, and IDO in the hippocampus. mRNA levels of M1 type markers iNOS and CD86 (D), and M2 type markers ARG1 and CD206 (E) in the hippocampus. (F and G) Western blot was performed to evaluate the effect of UB on the protein levels of Iba1, IDO, SIRT1, and FOXO1 in the hippocampus. *n* = 6–7 per group. ^*#*^ *p* < 0.05, ^*##*^ *p* < 0.01, ^*###*^ *p* < 0.001 versus the Vehicle + Vehicle group or LPS + Vehicle group.

In depression research, the LPS‐induced model is a commonly acknowledged and frequently used animal model [49, 50]; therefore, we evaluated the antidepressant activity and neuroinflammation response of UB using this model (Figure 2A). As expected, the results showed that the amount of sucrose preference measured in the LPS‐injected group was markedly less compared with the control group (*p* < 0.0010). After UB treatment, an obvious increase in sucrose preference percentage was found in the UB group contrasted with the LPS group (*p* = 0.0385) (Figure 2B; stress: *F* (1, 24) = 85.8700, *p* < 0.0010, treatment: *F* (1, 24) = 7.4500, *p* = 0.0117, interaction: *F* (1, 24) = 1.8020, *p* = 0.1920). In the TST, compared to the control group, the LPS group mice exhibited a prolonged total immobility time (*p* < 0.0010). However, the UB group mice had a decreased immobility time relative to the LPS group (*p* = 0.0017) (Figure 2B; stress: *F* (1, 24) = 14.4900, *p* < 0.0010, treatment: *F* (1, 24) = 6.2890, *p* = 0.0193, interaction: *F* (1, 24) = 11.6600, *p* = 0.0023). In the locomotor test, there was no obvious difference in the total distance among the groups (Figure 2B; stress: *F* (3, 24) = 0.7394, *p* = 0.5390, treatment: *F* (14, 336) = 26.6400, *p* < 0.0010, interaction: *F* (42, 336) = 1.2780, *p* = 0.1246; total distance: stress: *F* (1, 24) = 0.2658, *p* = 0.6108, treatment: *F* (1, 24) = 0.0089, *p* = 0.9256, interaction: *F* (1, 24) = 1.9430, *p* = 0.1761).

Next, we assessed the effect of UB after LPS stimulation on the gene expression of inflammatory factors. There was a marked rise of TNF‐α, IL‐1α, IL‐1β, and IL‐6 and IDO levels in the LPS group (*p* < 0.0010, *p* < 0.0010, *p* < 0.0010, *p* = 0.0190, *p* = 0.0308), which was relieved by the UB injection (*p* = 0.0015, *p* = 0.0027, *p* < 0.0010, *p* = 0.0120, *p* = 0.0261) (Figure 2C; TNF‐α: stress: *F* (1, 20) = 61.2700, *p* < 0.0010, treatment: *F* (1, 20) = 9.2170, *p* = 0.0065, interaction: *F* (1, 20) = 10.1200, *p* = 0.0047; IL‐1α: stress: *F* (1, 20) = 27.4800, *p* < 0.0010, treatment: *F* (1, 20) = 8.5690, *p* = 0.0083, interaction: *F* (1, 20) = 8.5220, *p* = 0.0085; IL‐1β: stress: *F* (1, 20) = 90.0400, *p* < 0.0010, treatment: *F* (1, 20) = 15.8400, *p* < 0.0010, interaction: *F* (1, 20) = 15.8100, *p* < 0.0010; IL‐6: stress: *F* (1, 20) = 3.8500, *p* = 0.0638, treatment: *F* (1, 20) = 5.1010, *p* = 0.0352, interaction: *F* (1, 20) = 6.9860, *p* = 0.0156; IDO: stress: *F* (1, 20) = 3.7200, *p* = 0.0681, treatment: *F* (1, 20) = 4.1540, *p* = 0.0550, interaction: *F* (1, 20) = 5.5610, *p* = 0.0287). Moreover, there were no obvious changes in IL‐4 and IL‐10 among the groups (Figure S2, IL‐4: stress: *F* (1, 20) = 2.0750, *p* = 0.1652, treatment: *F* (1, 20) = 0.2716, *p* = 0.6080, interaction: *F* (1, 20) = 0.0027, *p* = 0.9590; IL‐10: stress: *F* (1, 20) = 0.0398, *p* = 0.8439, treatment: *F* (1, 20) = 5.6280, *p* = 0.0278, interaction: *F* (1, 20) = 0.1395, *p* = 0.7127). Meanwhile, the mRNA levels of iNOS and CD86 in the hippocampus of the CUS‐stimulated mice were notably elevated compared to those of the control mice and decreased in UB‐treated mice (*p* = 0.0065 and *p* = 0.0355) (Figure 2D; iNOS: stress: *F* (1, 20) = 14.1700, *p* = 0.0012, treatment: *F* (1, 20) = 7.2070, *p* = 0.0143, interaction: *F* (1, 20) = 6.7720, *p* = 0.0170; CD86: stress: *F* (1, 20) = 94.1300, *p* < 0.0010, treatment: *F* (1, 20) = 4.0770, *p* = 0.0571, interaction: *F* (1, 20) = 4.7190, *p* = 0.0420). The results also indicated that UB protected the reduced mRNA levels of ARG1 but not CD206 induced by CUS treatment (Figure 2E; ARG1: *p* = 0.5983, *p* = 0.0100; stress: *F* (1, 20) = 0.6151, *p* = 0.4421, treatment: *F* (1, 20) = 6.0290, *p* = 0.0234, interaction: *F* (1, 20) = 6.5770, *p* = 0.0185; CD206: *p* = 0.0268, *p* = 0.8831; stress: *F* (1, 20) = 18.6300, *p* < 0.0010, treatment: *F* (1, 20) = 1.2050, *p* = 0.2855, interaction: *F* (1, 20) = 0.0038, *p* = 0.9509). After that, we evaluated the Iba1 expression levels to assess the microglia activation condition. The results showed an augmentation in the Iba1 and IDO protein in the LPS mice (*p* = 0.0193 and *p* = 0.0162), and UB restored this upregulation (*p* = 0.0144 and 0.0020) (Figure 2F,G; Iba1: stress: *F* (1, 20) = 1.4720, *p* = 0.2391, treatment: *F* (1, 20) = 1.9680, *p* = 0.1760, interaction: *F* (1, 20) = 11.4300, *p* = 0.0030; IDO: stress: *F* (1, 20) = 5.6920, *p* = 0.0270, treatment: *F* (1, 20) = 13.6800, *p* = 0.0014, interaction: *F* (1, 20) = 5.3940, *p* = 0.0309). The same phenomenon was also observed for the protein levels of SIRT1 and FOXO1 in the hippocampus (Figure 2F,G; SIRT1: *p* < 0.0010, *p* = 0.0350; stress: *F* (1, 20) = 18.5600, *p* < 0.0010, treatment: *F* (1, 20) = 1.7670, *p* = 0.1988, interaction: *F* (1, 20) = 8.2530, *p* = 0.0094; FOXO1: *p* < 0.0010, *p* = 0.0431; stress: *F* (1, 20) = 27.3800, *p* < 0.0010, treatment: *F* (1, 20) = 3.5220, *p* = 0.0752, interaction: *F* (1, 20) = 4.7710, *p* = 0.0401).

### 3.3 UB Reduces the Cytotoxicity and Apoptosis in HT22 Cells Treated by the Cellular Supernatant From LPS Incubated BV2 Cells

**FIGURE 3:**
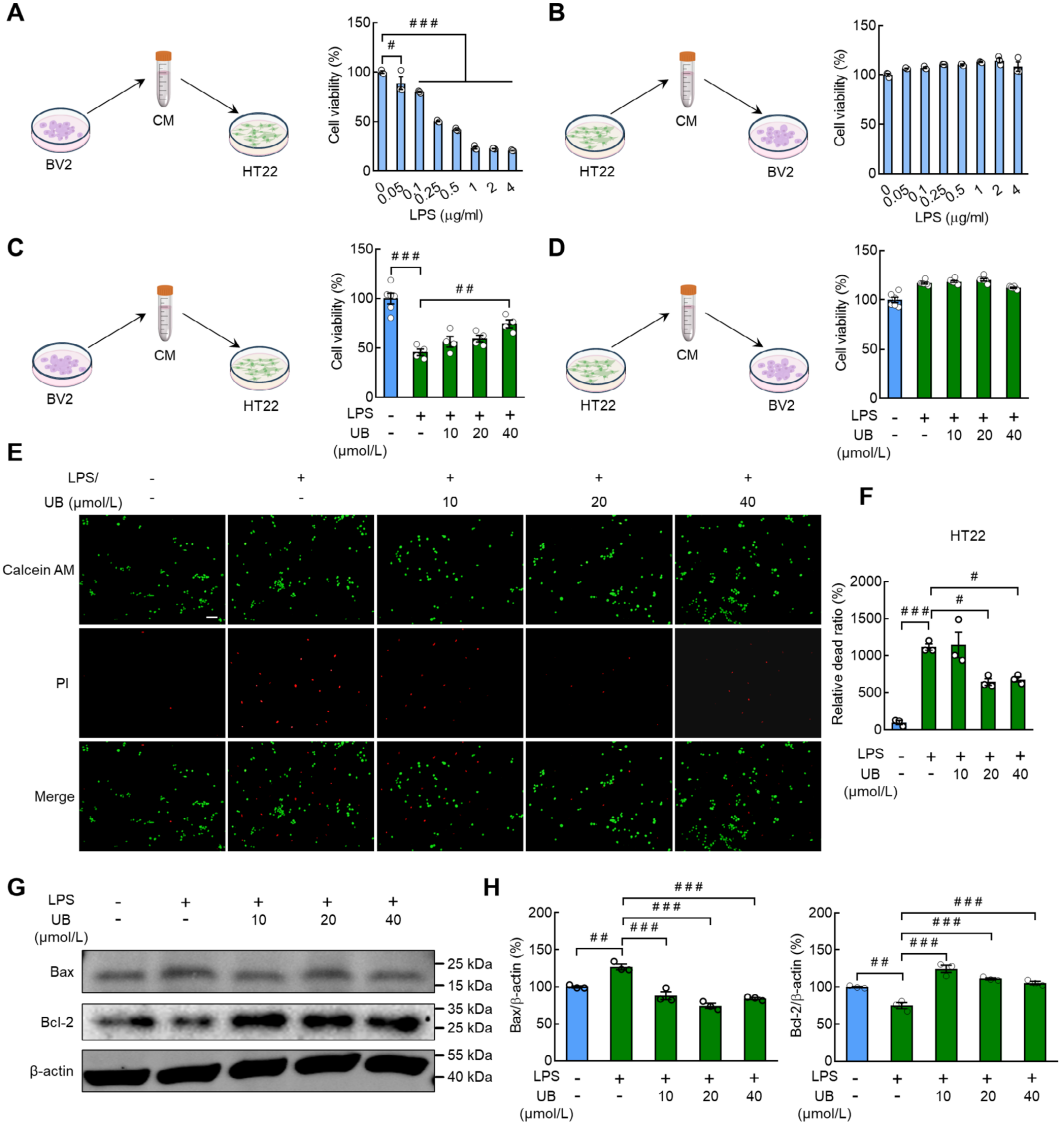
The effect of conditional medium (CM) from BV2 cells treated with LPS or/and UB on the proliferation and cell death of HT22 cells. Cell viability assay of the conditional medium collected from the BV2 (A) or HT22 (B) cells with LPS treatment. After treating BV2 or HT22 cells with 0.5 ng/mL LPS for 24 h, the medium supernatant was collected as conditional medium and HT22 or BV2 cells were cultured with conditional medium for 24 h, and then the cell viability was detected using CCK8 assay. Cell viability assay of the conditional medium collected from the BV2 (C) or HT22 (D) cells with LPS or/and UB treatment. (E and F) Live and dead cell double staining assay. BV2 cells were pretreated with UB (0, 10, 20, 40 μmol/L) for 2 h, then treated with LPS (0.5 μg/mL) for 24 h, followed by collecting the medium as conditional medium. HT22 cells were incubated with conditional medium for 24 h, and detected the ratio of live and dead cells by staining the live cells (green) with calcein AM and dead cells (red) with PI. Scale bar, 100 μm. (G and H) The protein levels of Bcl‐2 and Bax of HT22 cells after incubating with conditional medium for 24 h. *n* = 3–4 per group. ^*#*^ *p* < 0.05, ^*##*^ *p* < 0.01, ^*###*^ *p* < 0.001 versus the control group without LPS and UB treatment, or group treated with LPS alone.

In the beginning, we evaluated the cytotoxicity of LPS in the BV2 and HT22 cells, and the data demonstrated that both of the cell lines were not sensitive to LPS treatment, with no changes in cell viability (Figure S3A, BV2: *F* (7, 16) = 5.5460, *p* = 0.0022; HT22: *F* (7, 16) = 3.9520, *p* = 0.0108). As there are abundant crossed communications between glial cells and neurons, we measured the cytotoxicity of cellular supernatant from LPS‐incubated BV2 cells in HT22 cells. Interestingly, we found that cell viability obviously decreased after the specific supernatant incubation in comparison with that in the control group (*p* = 0.0262 and *p* < 0.0010) (Figure 3A, *F* (7, 16) = 177.9000, *p* < 0.0010), with decreased cell numbers and attenuated contact among cells (Figure S3B). In contrast, the cellular supernatant from LPS‐incubated HT22 cells exerted no virulent effect in BV2 cells (Figure 3B; *F* (7, 16) = 5.2630, *p* = 0.0029 and Figure S3C). In the following step, we purposed to test the impacts of UB on the LPS‐induced cellular reactions in BV2 cells. While UB itself demonstrated no cytotoxicity (Figure S4A, *F* (4, 15) = 2.2340, *p* = 0.1142), the results demonstrated a beneficial effect of UB against the attenuated cell viability induced by supernatant from LPS/BV2 cells in the HT22 cells (Figure 3C, *F* (4, 17) = 23.1600, *p* < 0.0010). On the contrary, there was a non‐significant effect of UB on the cell viability of BV2 induced by supernatant from LPS/HT22 cells (Figure 3D, *F* (4, 17) = 27.9400, *p* < 0.0010) under the safety concentrations without cytotoxicity (Figure S4B, *F* (4, 15) = 0.4790, *p* = 0.7509). Next, we carried out a Calcein AM/PI cellular toxicity assay for visualization of the extent of cell injury, and the results indicated that UB reversed the increased dead ratio for HT22 cells treated with the supernatant from LPS/BV2 cells (Figure 3E,F; *F* (4, 10) = 25.3600, *p* < 0.0010). Finally, we evaluated the apoptotic activity by monitoring the protein levels of Bcl‐2 and Bax and observed that the abnormal elevation of Bax and suppression of Bcl‐2 was restored by UB (Figure 3G,H, Bax: *F* (4, 10) = 36.3200, *p* < 0.0010; Bcl‐2: *F* (4, 10) = 34.1300, *p* < 0.0010).

### 3.4 Effects of UB on the LPS‐Induced Neuroinflammation, Microglial M1/M2 Polarization, and ROS Reactivity in BV2 Cells

**FIGURE 4:**
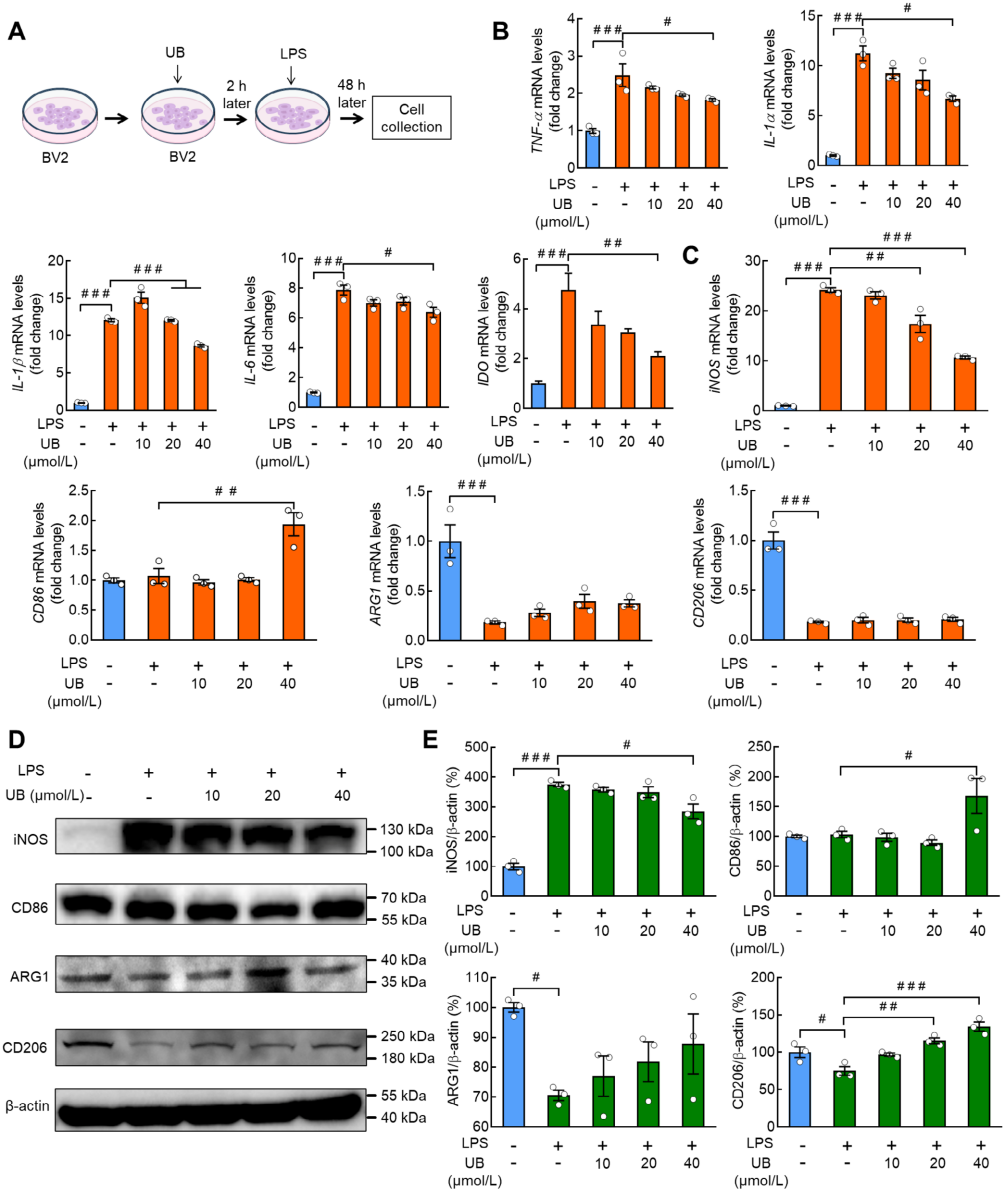
UB inhibits the production of proinflammatory cytokines and M1 polarization in LPS‐stimulated BV2 cells. (A) Experimental diagram. After pretreating with UB (0, 10, 20, 40 μmol/L) for 2 h, BV2 cells were treated with LPS (0.5 μg/mL) for 24 h. RT‐PCR was performed to measure the mRNA levels of inflammatory factors TNF‐ɑ, IL‐1ɑ, IL‐1β, IL‐6, and IL‐10, and IDO. (B) Polarization‐related markers iNOS, CD86, ARG1, and CD206 (C) in BV2 cells. (D‐E) Western blot was performed to detect the protein levels of iNOS, CD86, Arg1, and CD206 in BV2 cells. *n* = 3 per group. ^*#*^ *p* < 0.05, ^*##*^ *p* < 0.01, ^*###*^ *p* < 0.001 versus the control group without LPS and UB treatment, or the group treated with LPS alone.

As we found that the UB treatment changed the cytotoxicity of supernatant from LPS/BV2 cells, its potential molecular mechanisms were further investigated (Figure 4A). First, we measured the proinflammation factors and observed that there was a significant increase of TNF‐α, IL‐1α, IL‐1β, IL‐6, and IDO after LPS incubation (*p* < 0.0010, *p* < 0.0010, *p* < 0.0010, *p* < 0.0010, *p* < 0.0010), which was recovered by UB (*p* = 0.0480, *p* = 0.0026, *p* < 0.0010, *p* = 0.0153, *p* = 0.0070) (Figure 4B; TNF‐α: *F* (4, 10) = 15.4900, *p* < 0.0010; IL‐1α: *F* (4, 10) = 40.9600, *p* < 0.0010; IL‐1β: *F* (4, 10) = 236.3000, *p* < 0.0010; IL‐6: *F* (4, 10) = 114.6000, *p* < 0.0010; IDO: *F* (4, 10) = 11.7600, *p* < 0.0010). Additionally, we tested the M1/M2 polarization condition and observed that LPS induced higher mRNA (*p* < 0.0010) and protein (*p* < 0.0010) levels of M1 marker iNOS, which was suppressed by UB (*p* < 0.0010 and *p* = 0.0125), accompanied by unchanged CD86 mRNA (*p* = 0.9872) and protein (*p* = 0.9998) levels in the LPS‐treated groups and increased levels in the UB groups (*p* = 0.0015 and *p* = 0.0482) (Figure 4C, iNOS mRNA: *F* (4, 10) = 121.4000, *p* < 0.0010; CD86 mRNA: *F* (4, 10) = 14.8800, *p* < 0.0010; Figure 4D,E, iNOS protein: *F* (4, 10) = 55.5300, *p* < 0.0010; CD86 protein: *F* (4, 10) = 5.2870, *p* = 0.0150). Nevertheless, UB application showed no effect on the decreased ARG1 (M2 marker) mRNA (*p* = 0.5165) and protein (*p* = 0.3539) levels after LPS stimulation (*p* < 0.0010 and *p* = 0.0407). We also found that although CD206 (M2 marker) mRNA (*p* < 0.0010) and protein (*p* = 0.0496) levels were suppressed by LPS, only the protein levels were rescued by UB (*p* = 0.9989 and *p* < 0.0010) (Figure 4C, ARG1 mRNA: *F* (4, 10) = 14.6200, *p* < 0.0010; CD206 mRNA: *F* (4, 10) = 71.4100, *p* < 0.0010; Figure 4D,E, ARG1 protein: *F* (4, 10) = 3.2210, *p* = 0.0608; CD206 protein: *F* (4, 10) = 17.0800, *p* < 0.0010).

**FIGURE 5:**
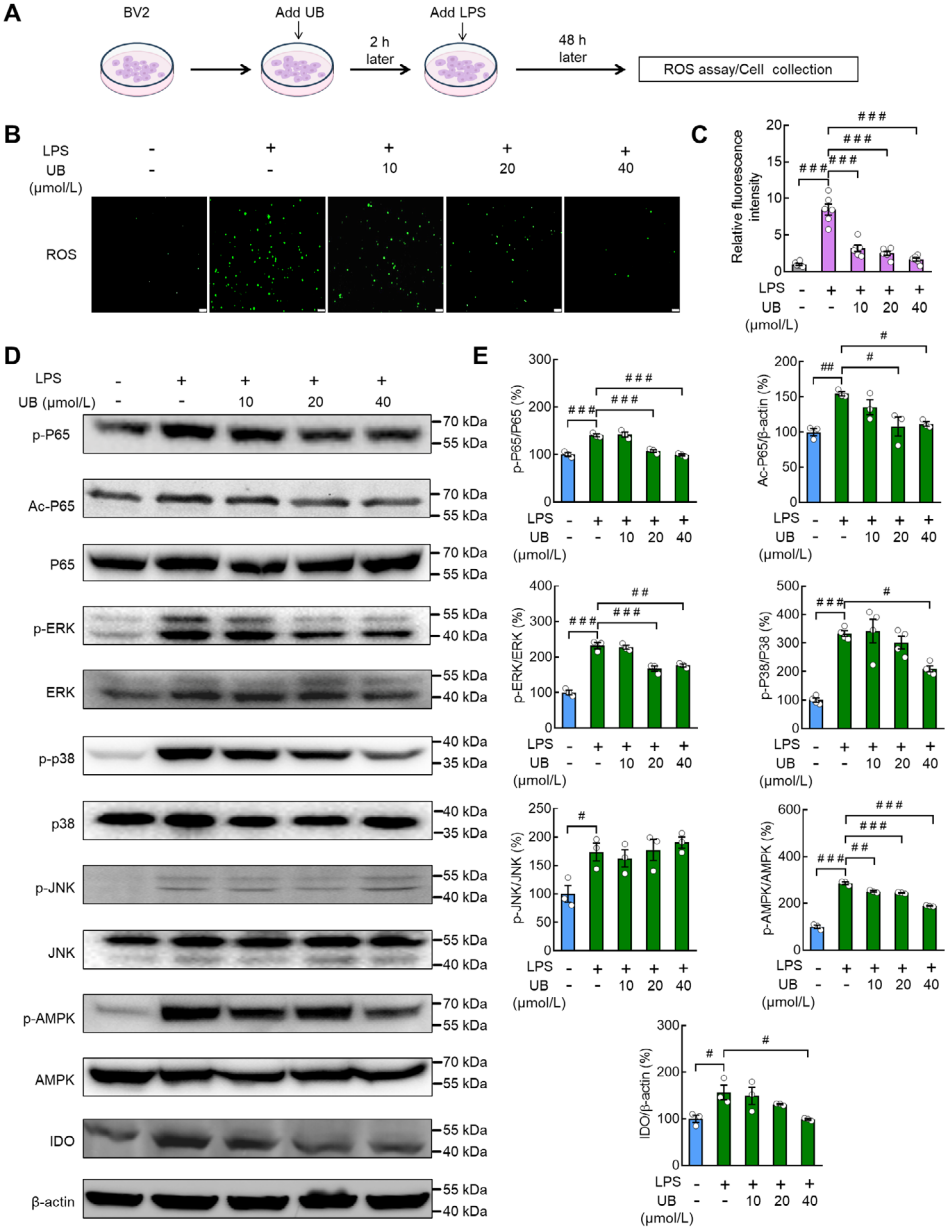
The effects of UB on the ROS and the phosphorylation levels of P65, ERK, P38, JNK, and AMPK of BV2 cells. (A) Experimental flow diagram. After pretreating with UB (0, 10, 20, 40 μmol/L) for 2 h, BV2 cells were treated with LPS (0.5 μg/mL) for 24 h. ROS and RNA extraction were performed. (B and C) Intracellular ROS levels were measured using the DCFH‐DA method. Scale bar, 50 μm. (D and E) Western blot was performed to detect the phosphorylation levels of P65, ERK, P38, JNK, and AMPK, the acetylation level of P65, and the expression levels of IDO of BV2 cells. *n* = 3 per group. ^*#*^ *p* < 0.05, ^*##*^ *p* < 0.01, ^*###*^ *p* < 0.001 versus the control group without LPS and UB treatment or the group treated with LPS alone.

Oxidative stress is an important player in microglial activity regulation [51]. Consequently, we tested the ROS activity (Figure 5A) and observed that the abnormal upregulation of ROS fluorescence levels (*p* < 0.0010) was attenuated by UB (*p* < 0.0010) (Figure 5B,C, *F* (4, 25) = 48.3900, *p* < 0.0010). Then some crucial signaling pathways related to neuroinflammation and ROS reactivity were monitored, and the LPS treatment induced an increase in p‐P65 (*p* < 0.0010), Ac‐P65 (*p* = 0.0064), p‐ERK (*p* < 0.0010), p‐p38 (*p* < 0.0010), p‐JNK (*p* = 0.0376), p‐AMPK (*p* < 0.0010), and IDO (*p* = 0.0352) levels, which were dramatically reversed by UB (*p* < 0.0010) except for p‐JNK (*p* < 0.0010, *p* < 0.0500, *p* < 0.0010, *p* = 0.0103, *p* > 0.0500, *p* < 0.0010, *p* = 0.0318) (Figure 5D,E, p‐P65: *F* (4, 10) = 37.3600, *p* < 0.0010; Ac‐P65: *F* (4, 10) = 7.3610, *p* = 0.0050; p‐ERK: *F* (4, 10) = 64.8800, *p* < 0.0010; p‐p38: *F* (4, 15) = 20.9400, *p* < 0.0010; p‐JNK: *F* (4, 10) = 5.5710, *p* = 0.0127; p‐AMPK: *F* (4, 10) = 261.6000, *p* < 0.0010; IDO: *F* (4, 10) = 5.5970, *p* = 0.0125).

### 3.5 Proteomics Analysis and Validation of the Cellular Supernatant Content From UB or LPS‐Treated BV2 Cells

**FIGURE 6:**
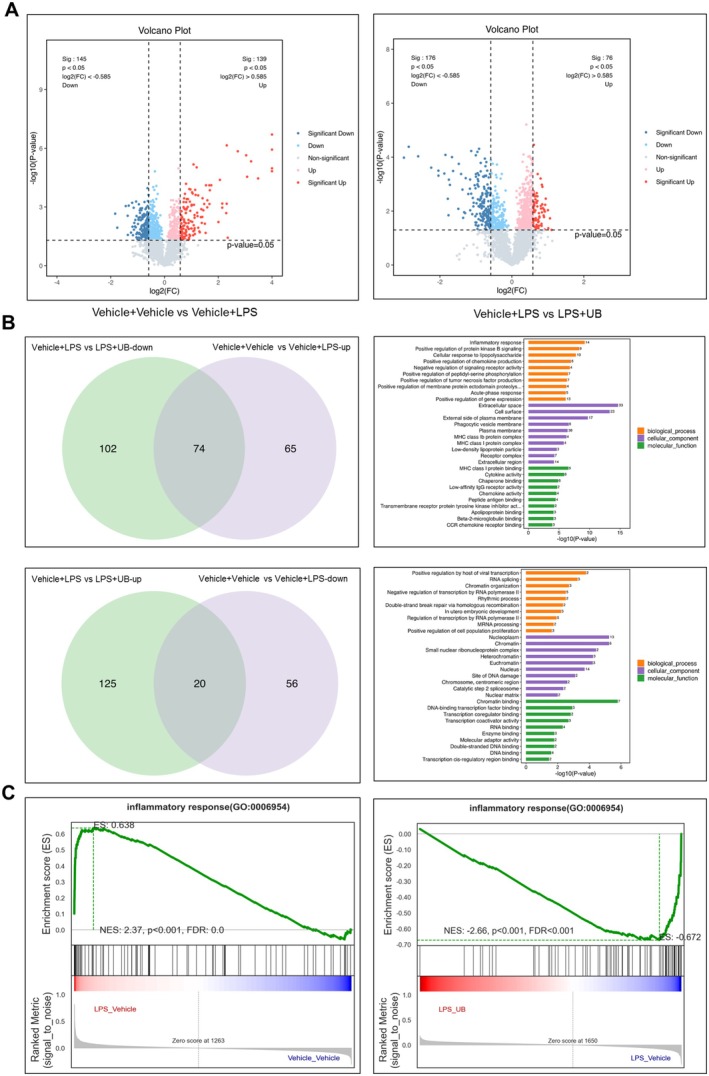
The effect of UB on the proteins secreted by BV2 cells was detected by Quantitative proteomics analysis. (A) Volcano map. (B) Venn map and KEGG analysis. (C) GSEA analysis.

In the next step, we explored the possible molecular changes under the LPS or UB‐treated BV2 cells. Quantitative proteomics analysis was performed on the supernatant from vehicle or LPS‐incubated BV2 cells and vehicle or UB and LPS‐incubated BV2 cells. As shown in the heatmap (Figure S5) and volcano plot (Figure 6A), LPS incubation induced 76 downregulated and 139 upregulated proteins, and UB treatment resulted in 176 downregulated and 145 upregulated proteins (Table S2). The overlap analysis demonstrated that the LPS‐induced 74 upregulated proteins were restored by UB treatment. GO and KEGG analyses were performed on the 74 intersection targets to evaluate their common biological functions and pathways. Functional enrichment analysis showed 140 cell components (CC), 2631 biological processes (BP), and 239 molecular functions (MF) enrichment items. These functions included multiple responses to inflammation, extracellular space, and cytokine (Figure 6B). The further overlap analysis also showed that the LPS‐induced 20 downregulated proteins were normalized by the UB treatment. GO functional enrichment analysis showed 140 cell components (CC), 2631 biological processes (BP), and 239 molecular functions (MF) enrichment items, which were mainly enriched in RNA splicing, nucleoplasm, and chromatin binding (Figure 6B). The inflammatory response was obvious and a remarkable aspect regarding the LPS or UB treatment (Figure 6C).

**FIGURE 7:**
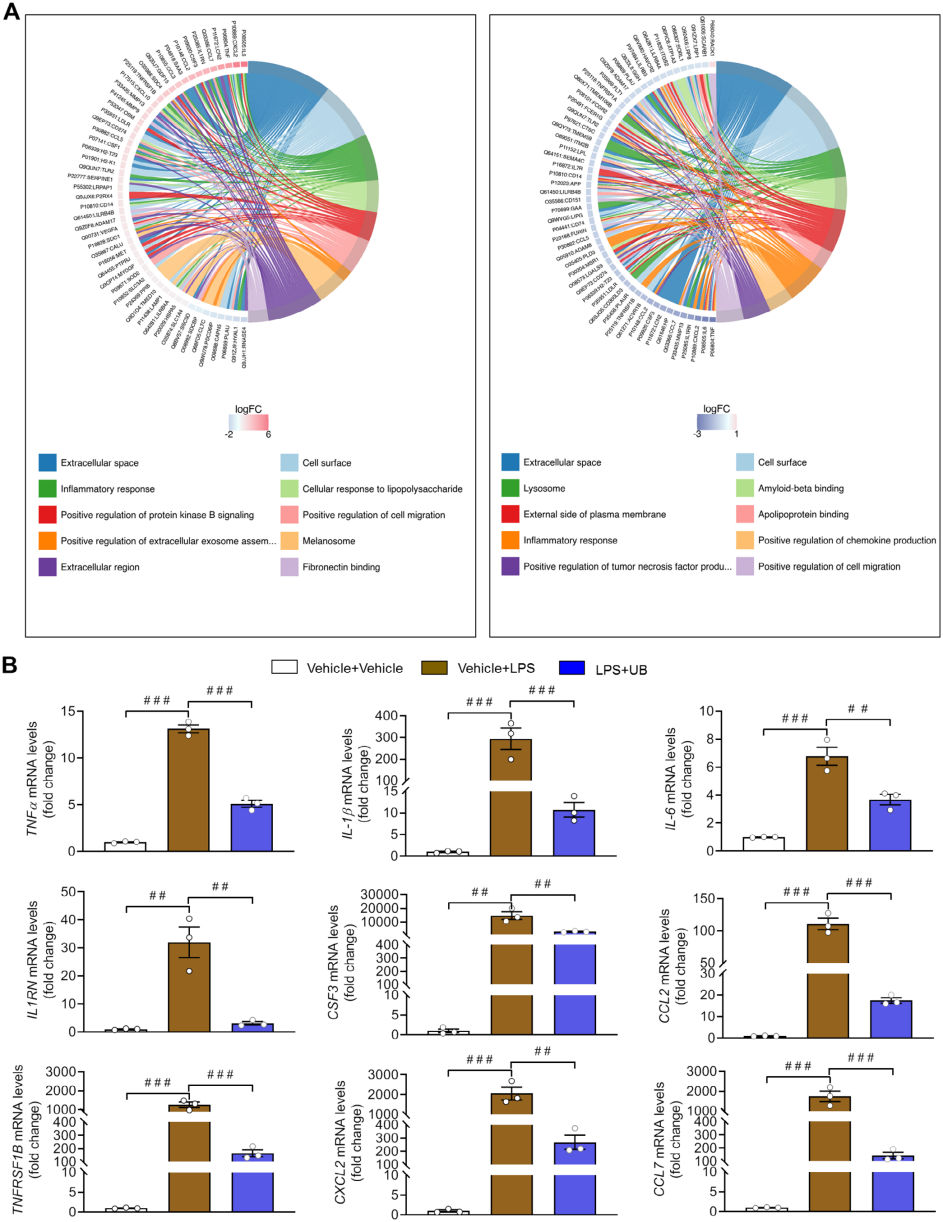
The effect of UB on the proteins secreted by BV2 cells. (A) KEGG enrichment analysis of chord diagrams. (B) The mRNA level of target genes. *n* = 3 per group. ^*##*^ *p* < 0.01, ^*###*^ *p* < 0.001 versus the Vehicle + LPS group or LPS + UB group.

The network analysis also verified these relevant changes and functional characteristics (Figure 7A). We conducted real‐time PCR quantitation to validate expression levels and some key factors in BV2 cells associated with inflammatory activities. We expected to find increased levels of TNF‐α, IL‐1β, IL‐6, IL‐1 receptor antagonist (IL1RN), colony stimulating factor 3 (CSF3), C‐C motif chemokine ligand (CCL2), TNF receptor superfamily member 1B (TNFRSF1B), and C‐X‐C Motif Chemokine Ligand 2 (CXCL2) after LPS application and the reversal effect of UB on these factors (Figure 7B, TNF‐α: *F* (2, 6) = 352.1000, *p* < 0.0010; IL‐1β: *F* (2, 6) = 33.6700, *p* < 0.0010; IL‐6: *F* (2, 6) = 45.3000, *p* < 0.0010; IL1RN: *F* (2, 6) = 29.7100, *p* < 0.0010; CSF3: *F* (2, 6) = 23.3400, *p* = 0.0015; CCL2: *F* (2, 6) = 133.0000, *p* < 0.0010; TNFRSF1B: *F* (2, 6) = 62.2200, *p* < 0.0010; CXCL2: *F* (2, 6) = 35.2100, *p* < 0.0010, CCL7: *F* (2, 6) = 39.6300, *p* < 0.0010).

### 3.6 Attenuation of Depression‐Like Behaviors Induced by the Cellular Supernatant Derived From LPS Incubated BV2 Cells Owing to UB

**FIGURE 8:**
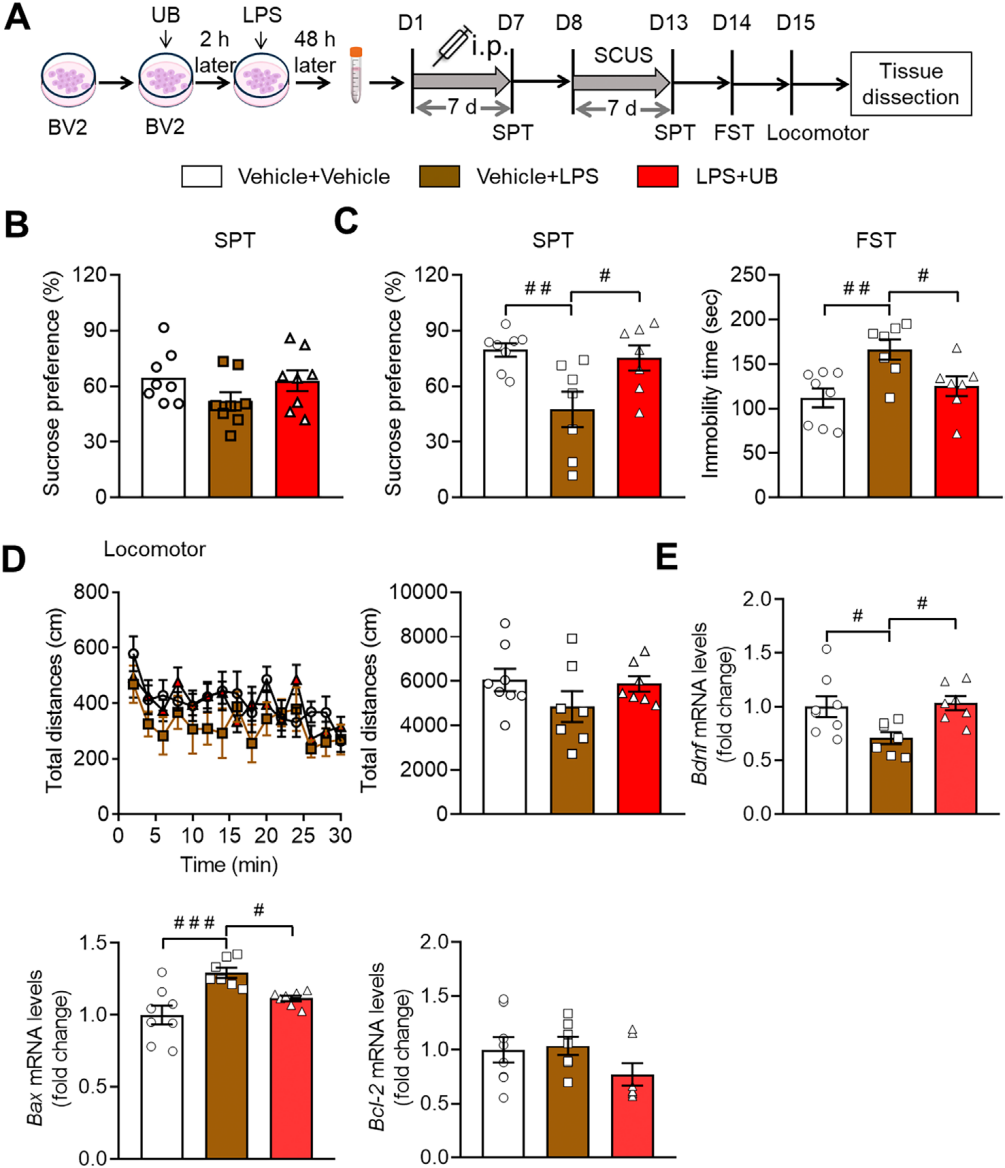
The effect of conditional medium from BV2 cells on the depressive behaviors in mice. (A) Scheme of the experimental procedure. (B) SPT. (C) SPT and FST. (D) Locomotor. (E) The mRNA expression levels of BDNF in the hippocampus. *n* = 7–8 per group. ^*#*^ *p* < 0.05, ^*##*^ *p* < 0.01, ^*###*^ *p* < 0.001 versus the LPS + Vehicle group, or LPS + UB group.

The next experiment was designed to measure the effect of cellular supernatant derived from LPS or UB‐incubated BV2 cells on depression‐related behaviors. WT mice were given the supernatant with or without LPS or UB by intraperitoneal injection for 7 days (Figure 8A). The SPT results showed no marked changes within the groups (Figure 8B, *F* (2, 21) = 1.7760, *p* = 0.1939). Then, these mice were subjected to a subthreshold CUS, which was adapted to test the stress susceptibility [41]. Interestingly, the results showed that LPS‐induced supernatant produced depression‐related behaviors with a decreased sucrose preference in SPT (*p* = 0.0091) and increased immobility in FST (*p* = 0.0060), which was restored by UB and LPS co‐treated supernatant (*p* = 0.0290 and *p* = 0.0452) (Figure 8C, SPT: *F* (2, 19) = 6.3810, *p* = 0.0076; FST: *F* (2, 19) = 6.6330, *p* = 0.0065). There was no difference in locomotor activity across the groups (Figure 8D, treatment: *F* (2, 19) = 1.4420, *p* = 0.2621; timepoints: *F* (14, 266) = 4.6640, *p* < 0.0010, interaction: *F* (28, 266) = 1.0270, *p* = 0.4323; total distance *F* (2, 19) = 1.4420, *p* = 0.2612).

Finally, the expression levels of BDNF, a crucial and important player in regulating the pathology of depression [52, 53], were found to decrease (*p* = 0.0382) and the apoptotic activity marker Bax was found to increase (*p* < 0.0010) in the LPS‐induced supernatant group, and UB application suppressed this abnormality (*p* = 0.0246 and *p* = 0.0440), while the expression levels of Bcl‐2 remain unchanged in these groups (Figure 8E, BDNF *F* (2, 19) = 5.1150, *p* = 0.0167; Bax: *F* (2, 19) = 9.9140, *p* = 0.0011; Bcl‐2: *F* (2, 19) = 1.8100, *p* = 0.1908).

### 3.7 Mediating Influence of the SIRT1‐FOXO1 Axis on the Activity of Cytotoxicity and Apoptosis in HT22 Cells Produced by the Cellular Supernatant of UB or LPS Treated BV2 Cells

**FIGURE 9:**
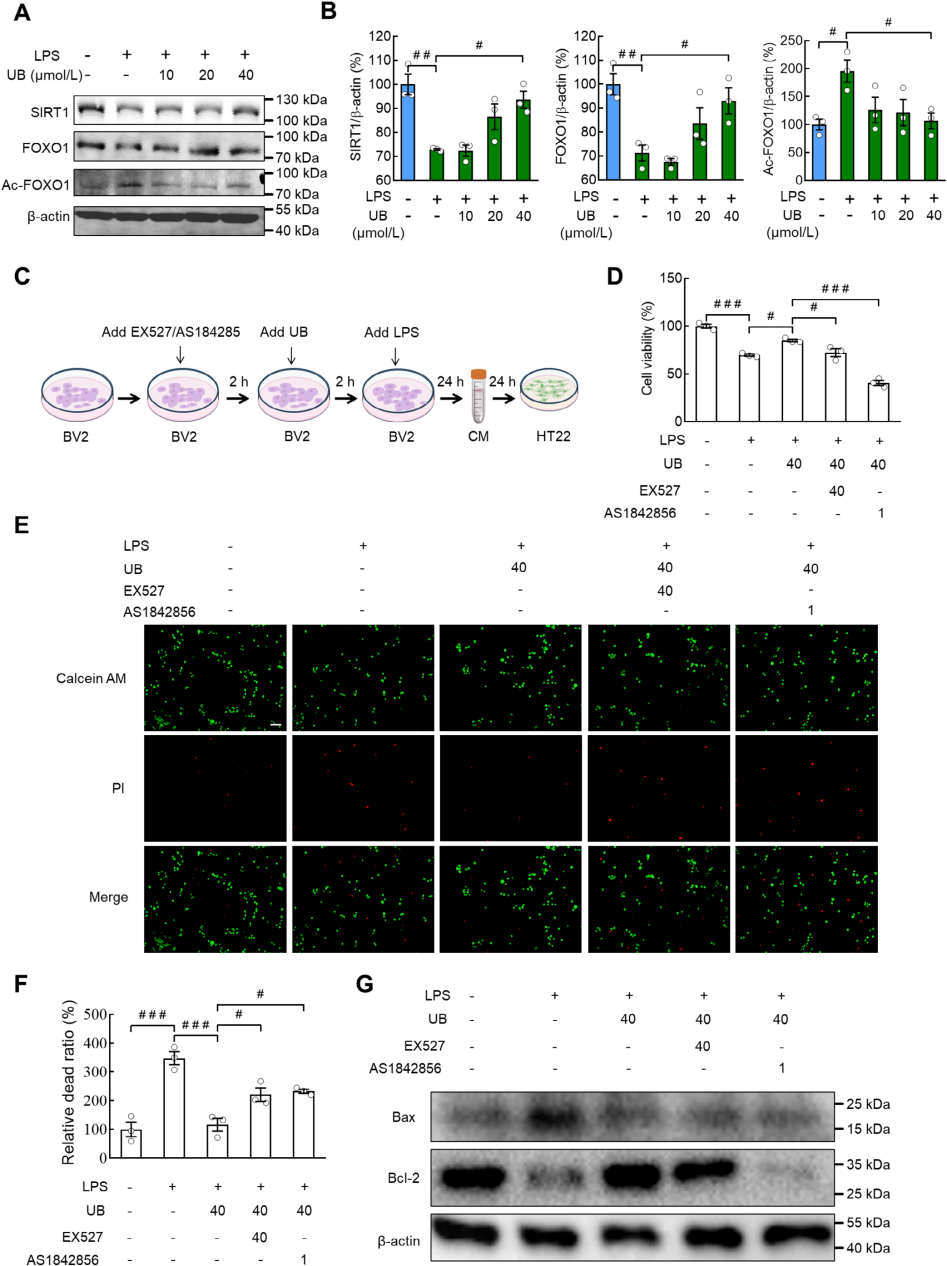
SIRT1 and FOXO1 inhibitors attenuated the function of UB. (A and B) Western blot was performed to detect the protein levels of SIRT1, FOXO1, and Ac‐FOXO1 in BV2 cells treated with LPS or UB. (C and D) Experimental flow diagram and CCK8 assay of HT22 cells after incubating with conditional medium from BV2 cells. (E and F) Live and dead cell double staining assay of HT22 cells. Scale bar, 100 μm. (G) Western blot was performed to detect the protein levels of Bax and Bcl‐2. *n* = 3 per group. ^*#*^ *p* < 0.05, ^*##*^ *p* < 0.01, ^*###*^ *p* < 0.001 versus the control groups.

To evaluate whether SIRT1 and FOXO1 were involved in the modulatory activity of UB in LPS‐treated BV2 cells, we measured the protein levels of SIRT1 and FOXO1. The results showed that the LPS‐induced reduction of SIRT1 (*p* = 0.0024) and FOXO1 (*p* = 0.0092) was recovered by UB (*p* = 0.0148 and *p* = 0.0463) (Figure 9A,B, SIRT1: *F* (4, 10) = 11.7700, *p* < 0.0010; FOXO1: *F* (4, 10) = 8.9860, *p* = 0.0024). The acetylation level of FOXO1 was enhanced by LPS treatment (*p* = 0.0303) and recovered by UB (*p* = 0.0449) (Figure 9A,B, *F* (4, 10) = 4.1710, *p* = 0.0305). Double immunostaining of SIRT1 or FOXO1 with Iba1 in BV2 cells indicated the increased trend of SIRT1 (Figure S6A,B, *F* (4, 10) = 134.7000, *p* < 0.0010) and FOXO1 (Figure S6C,D *F* (4, 10) = 95.5600, *p* < 0.0010) levels accompanied by the decreased Iba1 levels (Figure S6, *F* (4, 10) = 29.8000, *p* < 0.0010 and *F* (4, 10) = 31.1400, *p* < 0.0010).

Next, we tested whether SIRT1 and FOXO1 mediated the inhibitory effect of UB in the cytotoxicity and apoptosis induced by supernatant from LPS/BV2 cells in the HT22 cells. SIRT1 inhibitor EX527 or FOXO1 inhibitor AS1842856 was selected, and the safety concentrations in BV2 and the HT22 cells were determined when compared with the vehicle (Figure S7A, BV2, EX527: *F* (6, 14) = 1.2540, *p* = 0.3381; AS1842856: *F* (5, 12) = 5.4800, *p* = 0.0074); Figure S7B, HT22, EX527: *F* (6, 14) = 2.7140, *p* = 0.0580; AS1842856: *F* (5, 12) = 4.6230, *p* = 0.0139. The results revealed that both the SIRT1 inhibitor EX527 and the FOXO1 inhibitor AS1842856 blocked the therapeutic activity of UB with recovered cell viability (Figure 9C,D, *F* (4, 10) = 77.2600, *p* < 0.0010), dead ratio (Figure 9E,F, *F* (4, 10) = 22.5300, *p* < 0.0010) and protein levels of Bcl‐2 and Bax in the HT22 cells (Figure 9G). Furthermore, we observed that the alleviation of the ROS response, along with the inflammation factors of TNF‐α, IL‐1β, and IL‐6, and IDO by UB was also blocked by EX527 or AS1842856 (Figure S8A, ROS: *F* (4, 10) = 36.1200, *p* < 0.0010; Figure S8B, TNF‐α: *F* (4, 10) = 51.9300, *p* < 0.0010; IL‐1β: *F* (4, 10) = 34.5400, *p* < 0.0010; IL‐6: *F* (4, 10) = 42.4400, *p* < 0.0010; IDO: *F* (4, 10) = 9.798, *p* = 0.0017).

### 3.8 Mediating Influence of Hippocampal SIRT1 in Microglia on the Antidepressant Role of UB in LPS‐Treated Mice

**FIGURE 10:**
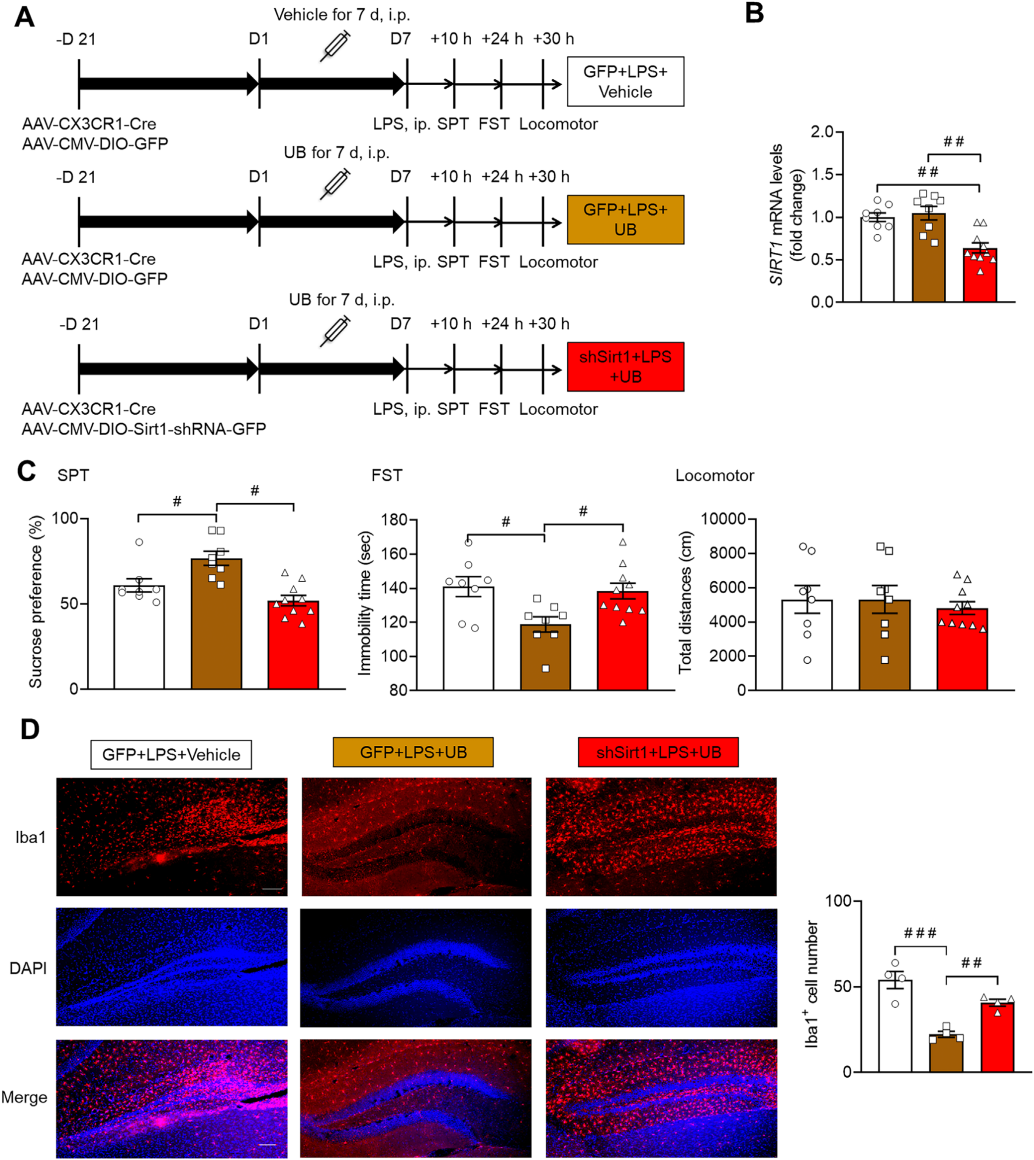
Knockdown SIRT1 in the hippocampus antagonized the antidepressant effects of UB. (A) Schematic diagram of virus injection and depression‐related behavior tests. (B) The mRNA levels of SIRT1 in the hippocampus. (C) SPT, FST, and locomotor tests. (D) Immunofluorescence test of Iba1 in the hippocampus. Scale bar, 100 μm. *n* = 4–8 per group. ^*#*^ *p* < 0.05, ^*##*^ *p* < 0.01, ^*###*^ *p* < 0.001 versus the GFP + LPS + Vehicle or shSIRT1 + LPS + UB group.

To further investigate the possible role of SIRT1 in the antidepressant activity of UB, we first conducted the small hairpin RNA‐mediated knockdown of SIRT1 in microglial cells of the hippocampus and tested its effect on the antidepressant activity of UB in the LPS‐treated mice model (Figure 10A). Our results showed that SIRT1 mRNA levels were dramatically suppressed by the SIRT1‐shRNA‐GFP virus (Figure 10B, *F* (2, 23) = 12.9700, *p* < 0.0010). Further exploration indicated that SIRT1 knockdown obviously blocked the antidepressant effects of UB in SPT (*F* (2, 23) = 11.9100, *p* < 0.0010) and FST (*F* (2, 23) = 5.5230, *p* = 0.0110), but there was no difference in the locomotor activity among the groups (*F* (2, 23) = 0.2079, *p* = 0.8138) (Figure 10C). Meanwhile, the increased number of Iba1 positive cells induced by LPS was also attenuated by SIRT1 knockdown (*F* (2, 9) = 23.2700, *p* < 0.0010) (Figure 10D).

## 4 Discussion

In the current investigation, we observed that UB exerted efficient therapeutic activities on depression‐like behaviors, suppressed microglia activation and neuroinflammation, balanced M1/M2 polarization, and restored the abnormal levels of SIRT1 and FOXO1 in the hippocampus. Furthermore, our findings indicated that UB reduced the cytotoxicity and apoptosis of HT22 cells and/or depression‐related behaviors induced by the cellular supernatant of LPS‐incubated BV2 cells via the SIRT1‐FOXO1 axis. Additionally, using proteomics analysis, we identified a series of inflammatory factors and chemokines in these cellular supernatant contents. Finally, we found that microglial SIRT1 mediates the antidepressant effect of UB.

A previous study suggested that the advantageous impacts of pomegranate are mainly focused on the urolithins derived from the colonic microbiota metabolites of ellagitannins [8]. UB also exhibits neuroprotective activity by inhibiting neuroinflammation in several brain areas such as the hippocampus, cortex, and substantia nigra in the LPS‐injected mice model, and there is a growing body of reports dealing with the mechanisms of UB, which may contribute to its effects [16]. The present study showed that UB showed antidepressant activity in both CUS and LPS depression models (Figures 1 and 2), which is the first to demonstrate the antidepressive effects of UB in two types of animal models, thus amplifying its therapeutic functions in neuropsychological diseases. Ample evidence reveals that microglia‐mediated neuroinflammation is a pivotal cause of depression, which is also considered a microglia‐associated disorder [19, 54]. Likewise, we observed that UB attenuated the enhancive activation of microglial cells and enhanced many proinflammatory factors' expression (Figure 4), which is in accordance with the results of other research [16, 17, 55]. The balance between microglia M1 and M2 polarization is a promising prospect for reflecting the inflammatory phenotypes and neuronal activities [56]. In the present study, UB demonstrated an obvious role by restraining M1 polarization and promoting M2 polarization in the hippocampus of depressed mice, which corresponded with decreased proinflammatory factors, such as TNF‐α, IL‐1, and IL‐6 (Figures 1 and 2). Nevertheless, the anti‐inflammatory factors (IL‐4 and IL‐10) remained unchanged after UB treatment. It was also interesting to find that UB only suppressed M1 polarization but had a slight effect on M2 polarization in LPS‐treated BV2 cells, with the different results showing decreased proinflammatory factors and increased anti‐inflammatory factors, respectively, which aligns with the findings of another study [16]. These variable mechanisms may be mainly derived from the diverse characters and complicated immunoreactions between in vitro and in vivo tissues.

Previous studies have suggested bi‐directionally in microglia–neuron communications, and microglia possess a considerable influence on multiple aspects of neuronal functions [19, 48]. Our results provide additional evidence for this opinion by showing the cytotoxicity of the cellular supernatant of LPS‐incubated BV2 cells in HT22 neuronal cells (Figure 3). In contrast, the absence of the influence of BV2 cells by the cellular supernatant of LPS‐incubated HT22 cells indicates the potential unidirectional effect between BV2 cells and HT22 neuronal cells in the hippocampus. Validating this hypothesis, we also observed the emergence of depression‐related behaviors after injecting supernatant from LPS‐incubated BV2 cells in mice (Figure 8). Both the cytotoxicity effects and abnormal depression‐related behaviors were dramatically attenuated by the UB application. Microglia activation usually establishes a proinflammatory environment, with the generation of many exocrine molecules, including cytokines, inflammasomes, and neurotransmitters [48].

Our proteomics analysis indicated 74 functional factors that mediate the protective or therapeutic effects of UB, mainly by reversing the inflammatory response (Figures 6 and 7). Among these proteins, we also identified some novel cytokines such as IL1RN, CSF3, CCL2, and CXCL2. Previous research identified IL1RN, a protein that binds to IL‐1 receptors, as a useful marker for evaluating susceptibility to Tourette's syndrome, schizophrenia, and the postnatal development of the brain [57, 58, 59]. CSF3, which belongs to the colony stimulating factor (CSF) family, can stimulate the formation of colonies of mature cells [60], which may contribute to the development, survival, and homeostasis of neurons and microglial cells [61]. CCL2 stood as the pioneering human CC chemokine that has been recognized [62]. The considerable role of CCL2 on the CNS has been noticed to be involved in various neurobiological processes, along with reduced synaptic transmission/plasticity and neuronal excitability, and has had functional consequences on depression [63, 64]. Neutrophil chemoattractant CXCL2 is a member of the CXC chemokine family. It was reported that CXCL2 exhibits multiple and significant physiological functions by its mediation of inflammation [65]. Moreover, CXCL2 participates in neuroinflammation and is responsible for neural injuries [66]. In general, these cytokines or chemokines have prominent functions in the nervous system. However, their role in depression and exact molecular candidates remains unclear; hence, further exploration is required.

Numerous studies have reported that SIRT1 takes part in the neuropathological conditions of mental disorders, including depression [17, 32, 67]. Recently, research investigated SIRT1 as a potential mediator of the antidepressant acts of arjunolic acid, exercise, and resveratrol in modulating microglial activities [67, 68, 69]. However, most of these studies were confined to neural reactions. FOXO1, one of the FOXO transcription factors, was reported to be positively correlated with depression‐related behaviors in our and other studies [35, 70]. Our previous study also proved that the SIRT1‐FOXO1 axis was excellent in mediating the inhibitory activity of UA glioblastoma progression [71]. Nevertheless, the effect of the SIRT1‐FOXO1 axis in microglial cells for depression still needs more attention, especially regarding its role in therapeutic agents for depression. Our results suggest causal relations between microglial SIRT1 or FOXO1 and therapeutic activity of UB in depression‐related behaviors and/or cytotoxicity and provide more evidence to expand the powerful actions of SIRT1 in the pathology and treatment of depression (Figure 9).

There are several restrictions in our research. First, only male mice were used. Given sex‐based differences in depression [72] and the fact that SIRT1 is a sexually dimorphic gene in depression [73], future research should also investigate these impacts in female mice. Second, subsequent investigations should examine the pharmacological parameters and molecular targeting sites of UB. Taken together, our research has established the antidepressant properties of UB and the molecular mechanism in microglial cells in vivo and in vitro, which is mediated by the SIRT1‐FOXO1 axis. We further show that the many cellular factors secreted from microglial cells served as functional mediators for the therapeutic effects of UB in LPS‐induced cytotoxicity and depression‐related behaviors. These findings expand our knowledge of the role of SIRT1 and future new therapeutic strategies for depression.

## Author Contributions

**Cuilan Liu** and **Di Zhao:** methodology, Investigation, Funding acquisition. **Guoxing Yu** and **HengWei Du:** methodology, Investigation. **Lihong Xu** and **Yifan Cao:** resources, Software. **Minghu Cui, Wentao Wang, Dan Wang, Jing Liu, Fantao Meng** and **Fengai Hu:** methodology, Resources. **Wei Li** and **Jing Du:** resources, Software, Writing – original draft, Data curation, Supervision, Project administration. **Chen Li:** writing – original draft, Data curation, Funding acquisition, Supervision, Project administration.

## Conflicts of Interest

The authors declare no conflicts of interest.

## Supporting information


Figure S1.

Table S1.

Table S2.

## Data Availability

The datasets used and/or analyzed during the current study are available from the corresponding author on reasonable request.
