# Supplementary material for: Alleviation of Microglia Mediating Hippocampal Neuron Impairments and Depression‐Related Behaviors by Urolithin B via the SIRT1‐FOXO1 Pathway
Source: CNS Neurosci Ther. 2025 Apr 16;31(4):e70379. doi: 10.1111/cns.70379 (PMC12000931; doi:10.1111/cns.70379)
Supplement: Supplementary file 1 — Figure S1. [file CNS-31-e70379-s003.doc]

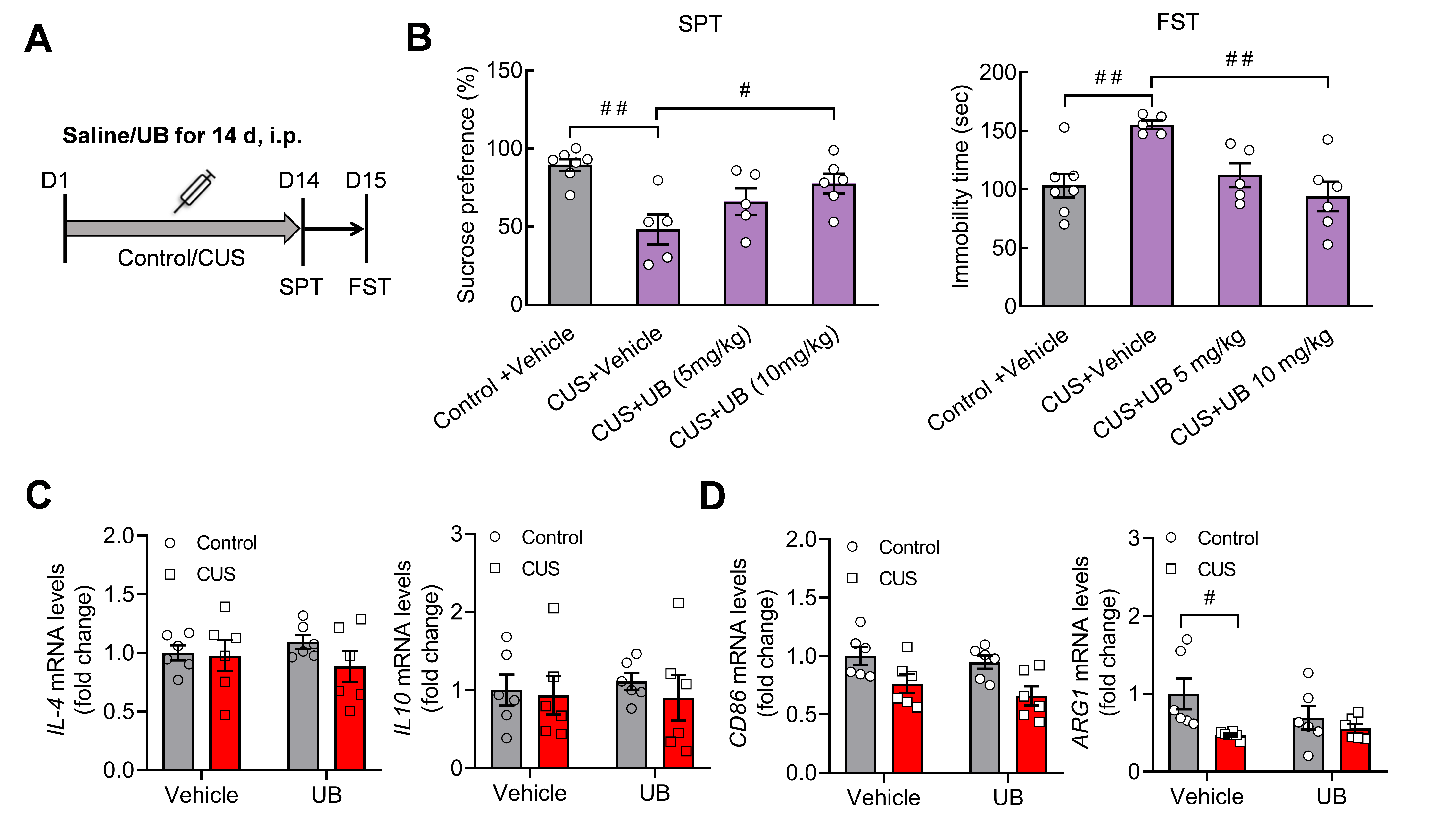


Fig. S1 The impact of varying doses of UB on depression-like behaviors induced by CUS, as well as its effects on the mRNA expression levels of IL-4, IL-10, CD86, and ARG1 in the hippocampus. (A) Schematic representation of the CUS procedure and treatments in mice. (B) The effects of UB at doses of 5 and 10 mg/kg on CUS-induced depression-like behaviors were assessed using the SPT and TST. SPT, sucrose preference test; FST, forced swimming test. (C) IL-4 and IL10. (D) CD86 and Arg1**.** *n* = 5-7 per group. *^#^p* < 0.05, *^# #^p* < 0.01.


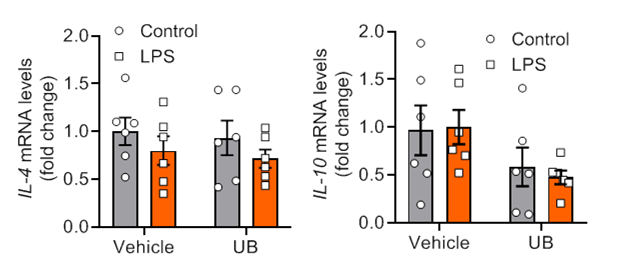


Fig. S2 The effect of UB on the IL-4 and IL10 mRNA levels in mice hippocampus in LPS-induced depression model. *n* = 6 per group


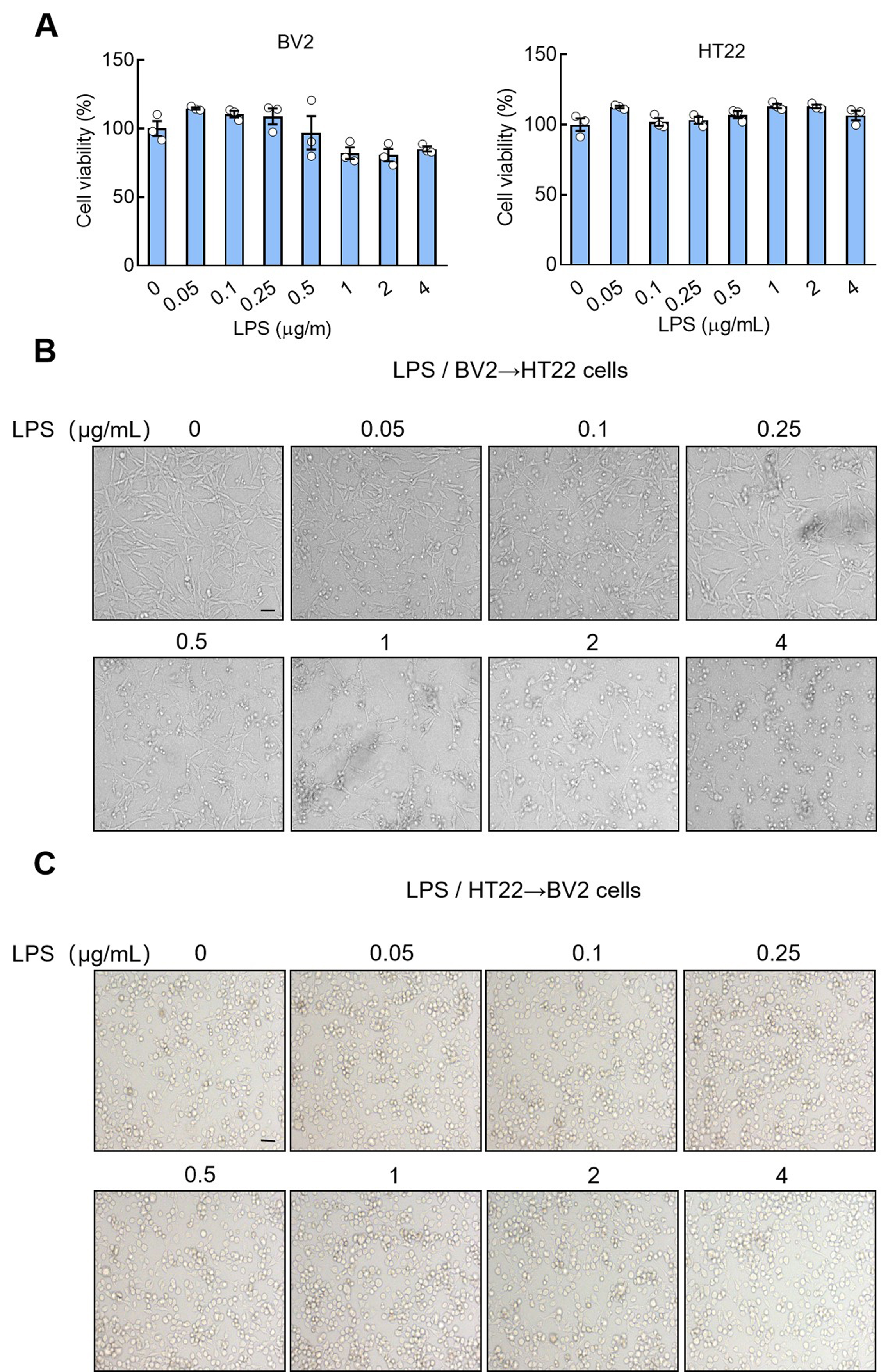


Fig. S3 The effect of LPS on the cell viability and morphology of BV2 and HT22. (A) CCK8 assay was performed to detect the cell viability of BV2 and HT22. (B) BV2 and HT22 cell were treated with different concentration of LPS for 24 h, then the conditional medium was collected. After treating HT22 and BV2 cells with conditional medium from BV2 and HT22 cells for 24 h, individually, and representative images of cell morphology. *n* = 3 per group.


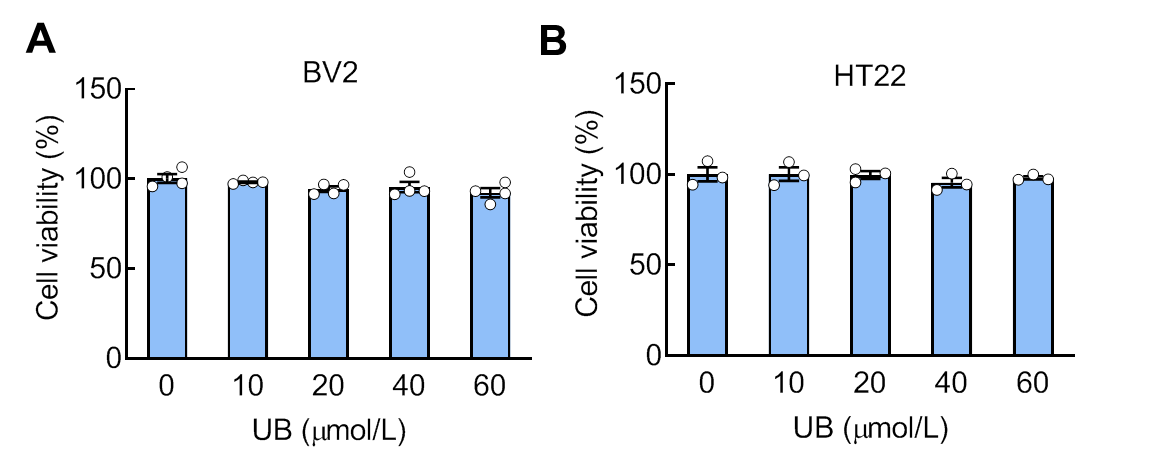


Fig. S4 Effects of different doses of UB on BV2 (A) and HT22 (B) cell viability. *n* = 3 per group.


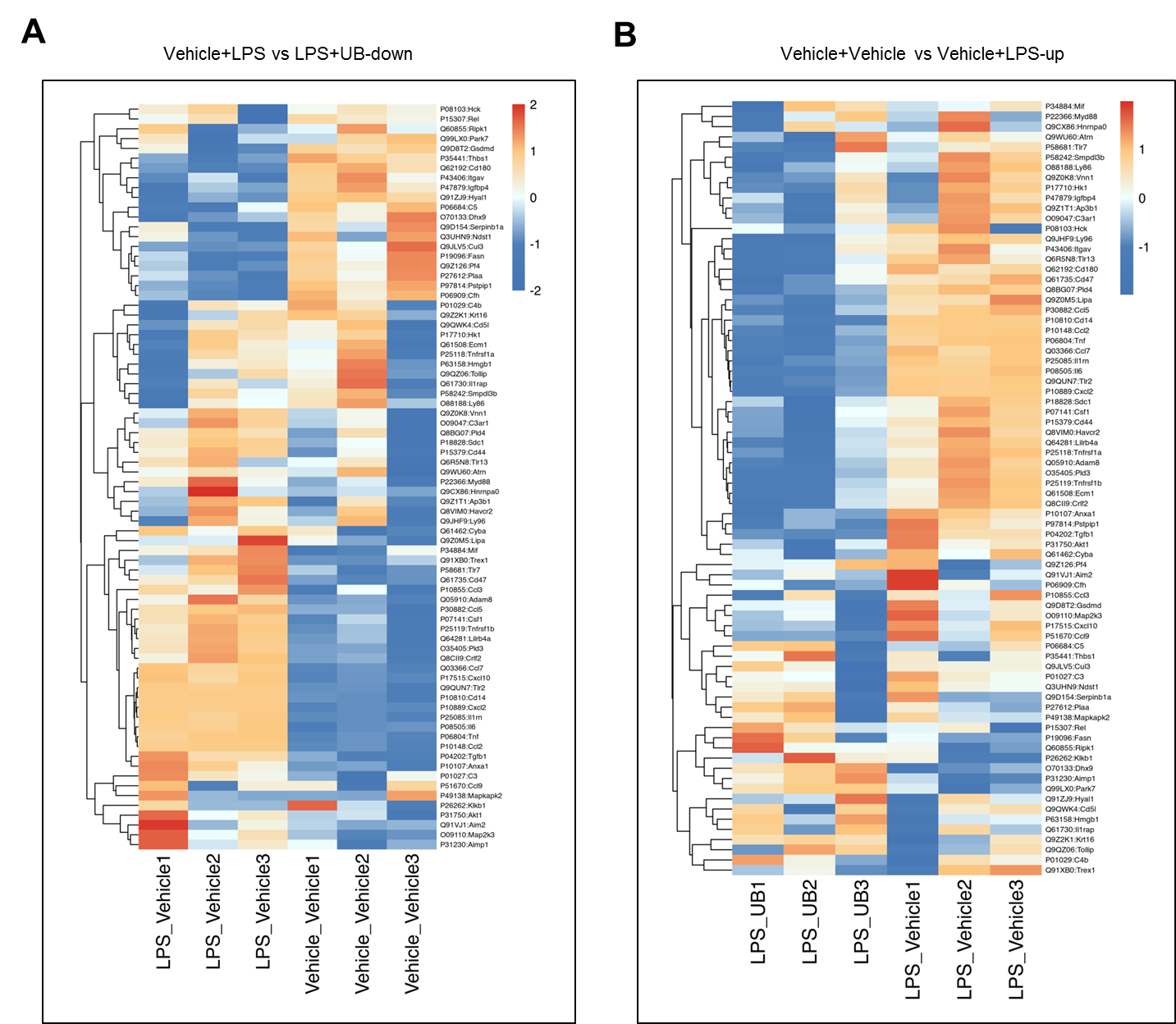


Fig. S5 The heatmap of the proteomics analysis on the supernatant from vehicle or LPS incubated BV2 cells and vehicle or UB treated LPS incubated BV2 cells.


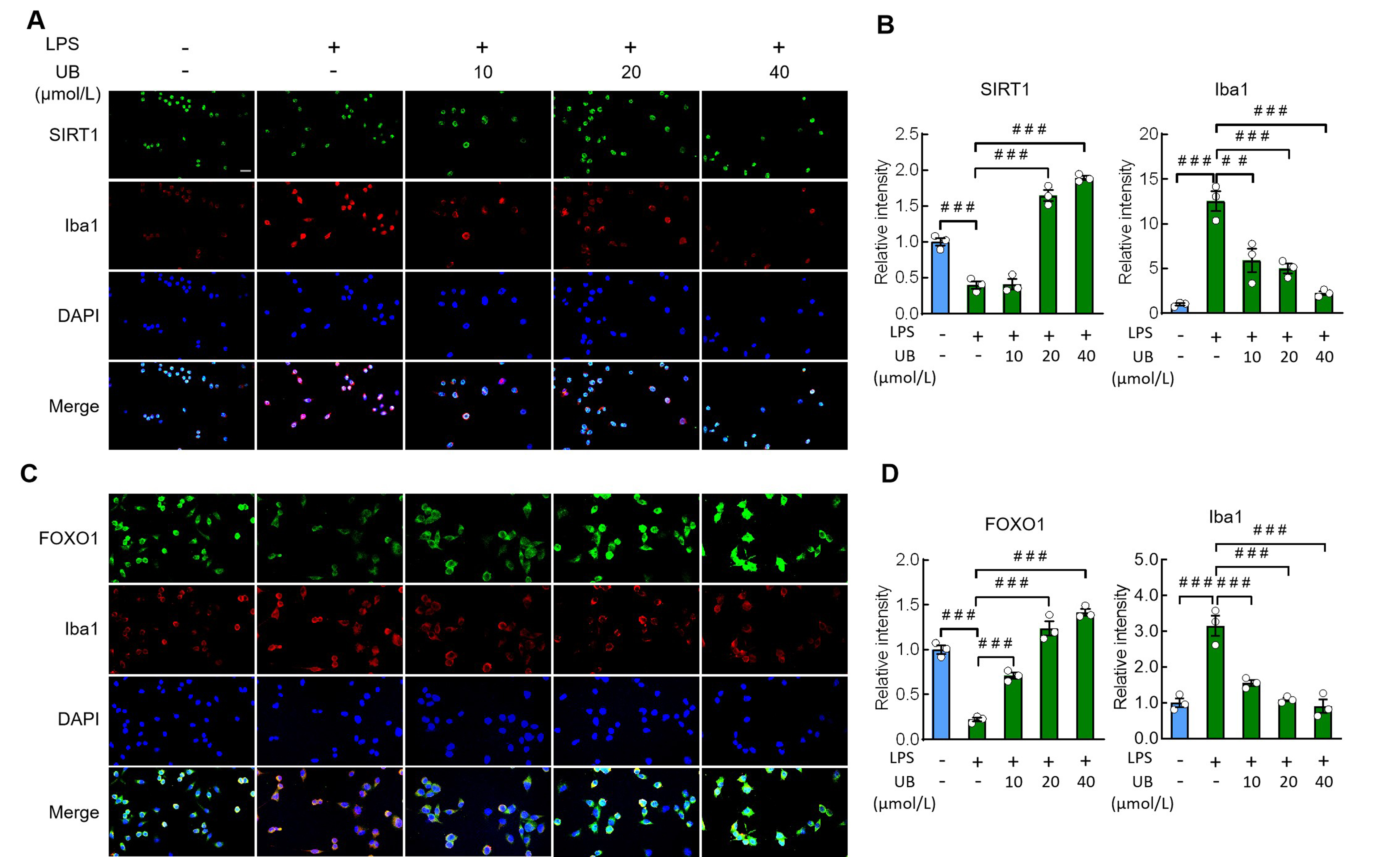


Fig. S6 Double immunostaining and intensity analysis of SIRT1 (A and B) or FOXO1 (C and D) with Iba1 in BV2 cells. Scale bar, 20 μm. *n* = 3 per group. ^# #^*p* < 0.01, ^# # #^*p* < 0.001 versus the control group.


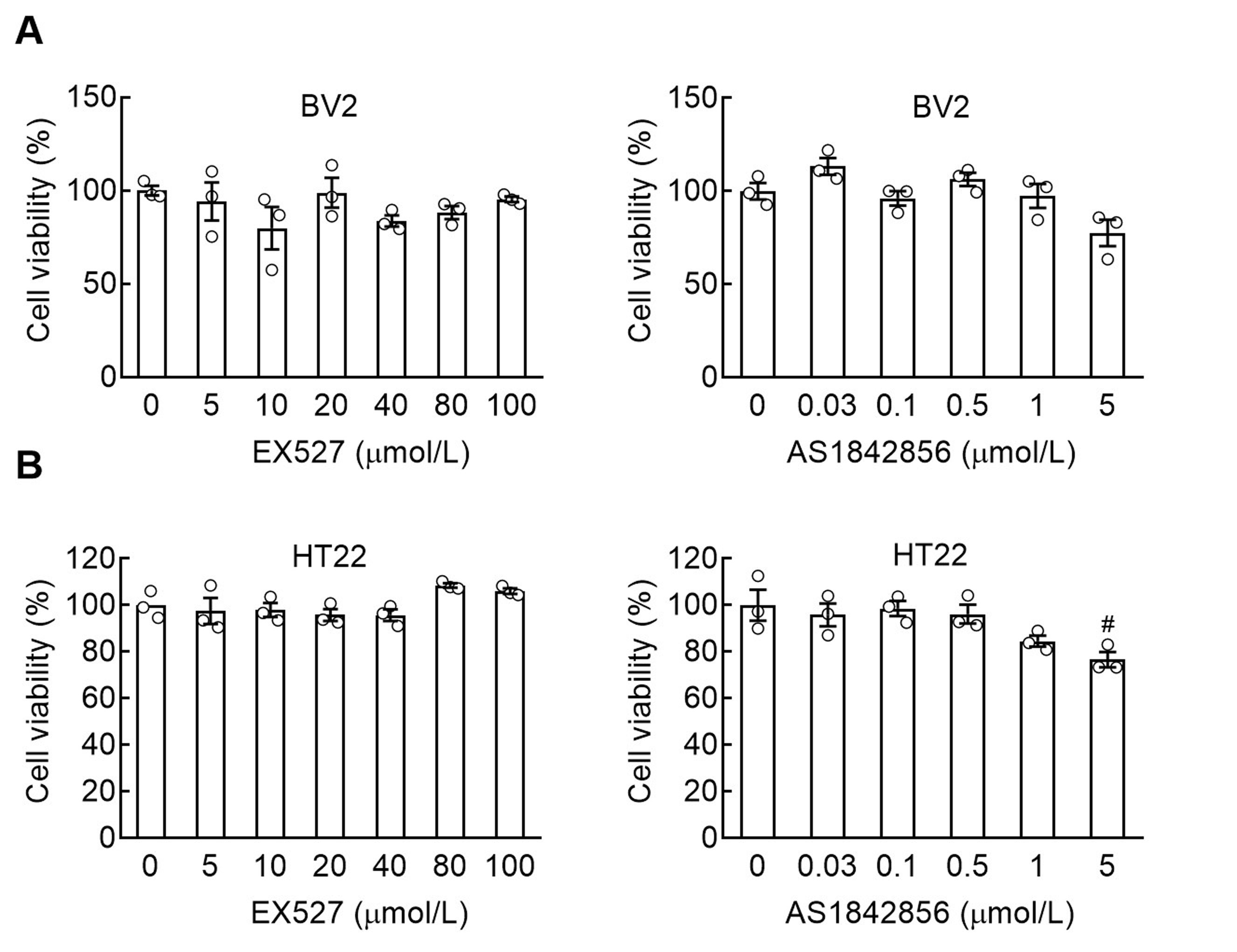


Fig. S7 The cytotoxicity of SIRT1 and FOXO1 inhibitors in BV2 cells. (A) Cell viability of BV2 cells after treatment with SIRT1 inhibitor EX527 or FOXO1 inhibitor. (B) Cell viability of HT22 cells after treatment with SIRT1 inhibitor EX527 or FOXO1 inhibitors AS1842856. *n* = 3 per group.


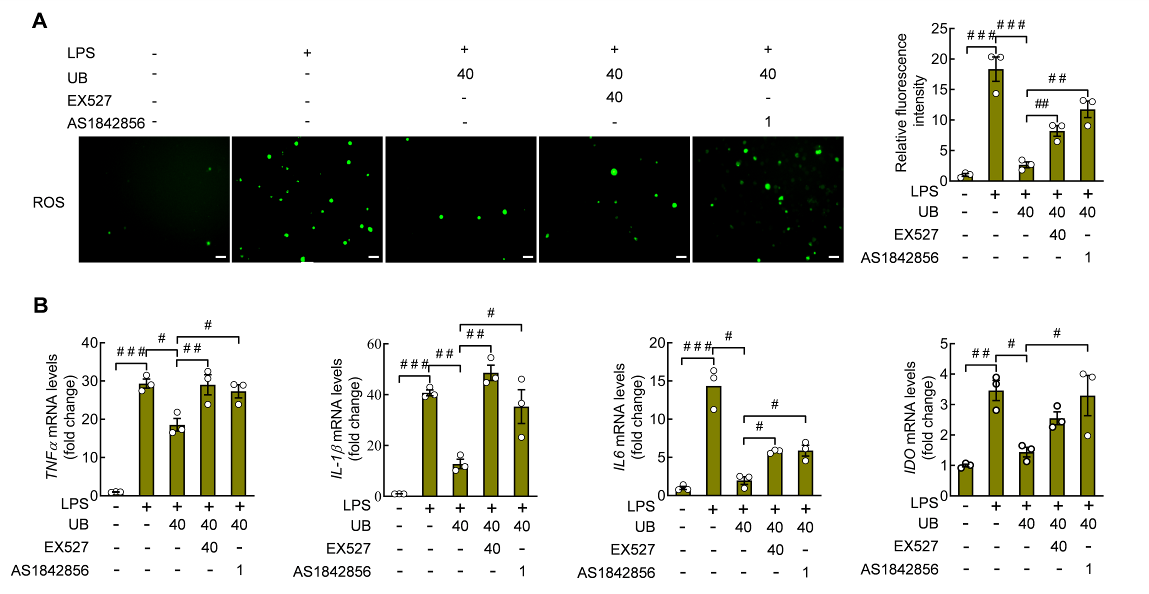


Fig. S8 SIRT1 and FOXO1 inhibitors attenuated the function of UB on ROS, secretion of inflammatory factors and IDO in BV2 cells. (A) ROS. (B) The mRNA expression levels of TNFɑ, IL-1β, IL6 and IDO. n = 3 per group. ^#^*p* < 0.05, ^# #^*p* < 0.01, ^# # #^*p* < 0.001 versus the control group.
