# Supplementary material for: Alleviation of Microglia Mediating Hippocampal Neuron Impairments and Depression‐Related Behaviors by Urolithin B via the SIRT1‐FOXO1 Pathway
Source: CNS Neurosci Ther. 2025 Apr 16;31(4):e70379. doi: 10.1111/cns.70379 (PMC12000931; doi:10.1111/cns.70379)
Supplement: Supplementary file 2 — Table S1. [file CNS-31-e70379-s002.docx]

Supplementary Table S1 Primer sequences for the analyzed genes

| Primer | Sequence |
| --- | --- |
| TNFɑ-Forward | CAGGCGGTGCCTATGTCTC |
| TNFɑ-Reverse | CGATCACCCCGAAGTTCAGTAG |
| IL6-Forward | TAGTCCTTCCTACCCCAATTTCC |
| IL6-Reverse | TTGGTCCTTAGCCACTCCTTC |
| IL-1β-Forward | GAAATGCCACCTTTTGACAGTG |
| IL-1β-Reverse | TGGATGCTCTCATCAGGACAG |
| IL4-Forward | GGTCTCAACCCCCAGCTAGT |
| IL4-Reverse | GCCGATGATCTCTCTCAAGTGAT |
| IL10-Forward | GCAGCTCTAGGAGCATGTGG |
| IL10-Reverse | CTTACTGACTGGCATGAGGATCA |
| CD206-Forward | CTCTGTTCAGCTATTGGACGC |
| CD206-Reverse | CGGAATTTCTGGGATTCAGCTTC |
| CD86-Forward | TGTTTCCGTGGAGACGCAAG |
| CD86-Reverse | TTGAGCCTTTGTAAATGGGCA |
| Arg1-Forward | CTCCAAGCCAAAGTCCTTAGAG |
| Arg1-Reverse | AGGAGCTGTCATTAGGGACATC |
| iNOS-Forward | CAGGAGGAGAGAGATCCGATTTA |
| iNOS-Reverse | GCATTAGCATGGAAGCAAAGA |
| CXCL2-Forward | CCAACCACCAGGCTACAGG |
| CXCL2-Reverse | GCGTCACACTCAAGCTCTG |
| CCL7-Forward | GCTGCTTTCAGCATCCAAGTG |
| CCL7-Reverse | CCAGGGACACCGACTACTG |
| IL1RN-Forward | GCTCATTGCTGGGTACTTACAA |
| IL1RN-Reverse | CCAGACTTGGCACAAGACAGG |
| CSF3-Forward | ATGGCTCAACTTTCTGCCCAG |
| CSF3-Reverse | CTGACAGTGACCAGGGGAAC |
| CCL2-Forward | TTAAAAACCTGGATCGGAACCAA |
| CCL2-Reverse | GCATTAGCTTCAGATTTACGGGT |
| TNFRSF1B-Forward | ACACCCTACAAACCGGAACC |
| TNFRSF1B-Reverse | AGCCTTCCTGTCATAGTATTCCT |
| IDO-Forward | CAAAGCAATCCCCACTGTATCC |
| IDO-Reverse | ACAAAGTCACGCATCCTCTTAAA |
| β-actin-Forward | GTTGGTTGGAGCAAACATC |
| β-actin-Reverse | CTTATTTCATGGATACTTGGAATG |
